# C–H functionalisation tolerant to polar groups could transform fragment-based drug discovery (FBDD)†

**DOI:** 10.1039/d1sc03563k

**Published:** 2021-09-01

**Authors:** Gianni Chessari, Rachel Grainger, Rhian S. Holvey, R. Frederick Ludlow, Paul N. Mortenson, David C. Rees

**Affiliations:** Astex Pharmaceuticals 436 Cambridge Science Park Cambridge CB4 0QA UK rachel.grainger@astx.com rhian.holvey@astx.com

## Abstract

We have analysed 131 fragment-to-lead (F2L) examples targeting a wide variety of protein families published by academic and industrial laboratories between 2015–2019. Our assessment of X-ray structural data identifies the most common polar functional groups involved in fragment-protein binding are: N–H (hydrogen bond donors on aromatic and aliphatic N–H, amides and anilines; totalling 35%), aromatic nitrogen atoms (hydrogen bond acceptors; totalling 23%), and carbonyl oxygen group atoms (hydrogen bond acceptors on amides, ureas and ketones; totalling 22%). Furthermore, the elaboration of each fragment into its corresponding lead is analysed to identify the nominal synthetic growth vectors. In ∼80% of cases, growth originates from an aromatic or aliphatic carbon on the fragment and more than 50% of the total bonds formed are carbon–carbon bonds. This analysis reveals that growth from carbocentric vectors is key and therefore robust C–H functionalisation methods that tolerate the innate polar functionality on fragments could transform fragment-based drug discovery (FBDD). As a further resource to the community, we have provided the full data of our analysis as well as an online overlay page of the X-ray structures of the fragment hit and leads: https://astx.com/interactive/F2L-2021/

## Abstract 2

An in depth meta analysis of 131 fragment-to-lead case-studies has shown the importance of synthetic methods that allow carbon-centred synthetic elaboration in the presence of polar pharmacophores.

## Graphical abstract

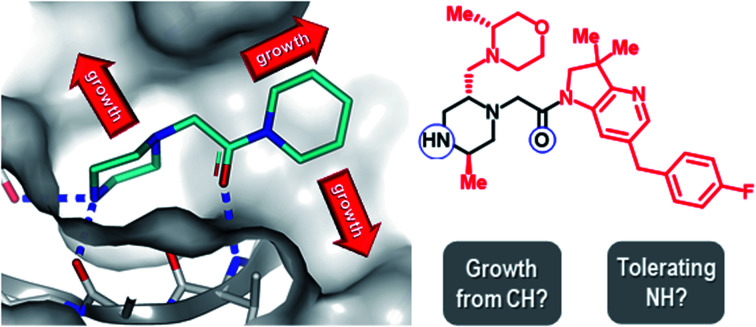

## 1. Introduction

Continued innovation in synthetic organic chemistry is of fundamental importance to the pharmaceutical industry. During early hits-to-leads and the lead-optimisation phase, the synthetic challenges presented by many drug-like molecules are often rate-limiting and this delay can ultimately impact the time it takes a drug to progress into the clinic and hence, patients for treatment.^1–3^ Medicinal chemists frequently face challenges related to finding suitable synthetic methods, tolerant to heterocycles and unprotected polar functionality. Invariably drug and drug-like molecules contain heteroatoms and polar groups key for protein binding, however these motifs often participate in undesirable side-reactions and transition-metal catalyst deactivation, unless protecting group strategies are employed.^3^

Recent analyses of common reaction types used in the pharmaceutical industry and disclosed in patents, suggest that an alarmingly high number of reactions performed by medicinal chemists are the protection/deprotection of heteroatoms.‡‡A 2011 analysis of the published output of three major pharmaceutical companies categorised reactions used in medicinal chemistry. Of the total reactions performed, 21.1% were some form of heteroatom protection/deprotection, with the majority for NH (39% protect, 46% deprotect) or CO_2_H (41% protect, 30% deprotect).^4^ A more recent 2016 analysis of U.S. patents disclosed from 1976–2015, showed that the number of different reaction types employed over this period had more than doubled, but that the proportion of protection/deprotection reactions was still very high (16.6% of the entire dataset).^5^^4,5^ This is inextricably linked to the challenges associated with synthesising hetero-atom-rich, drug-like molecules and illustrates the continuing need for expanding the traditional medicinal chemistry toolbox to include new methodologies. Some examples of note include: protecting-group free synthesis, biocatalysis, photoredox-catalysed transformations, electrochemistry, C–H bond functionalisation and late-stage functionalisation.^2,3,6–8^ By engaging in these burgeoning areas of cutting-edge synthesis, productive collaborations between academia and industry can be realised.^9^

**Fig. 1:**
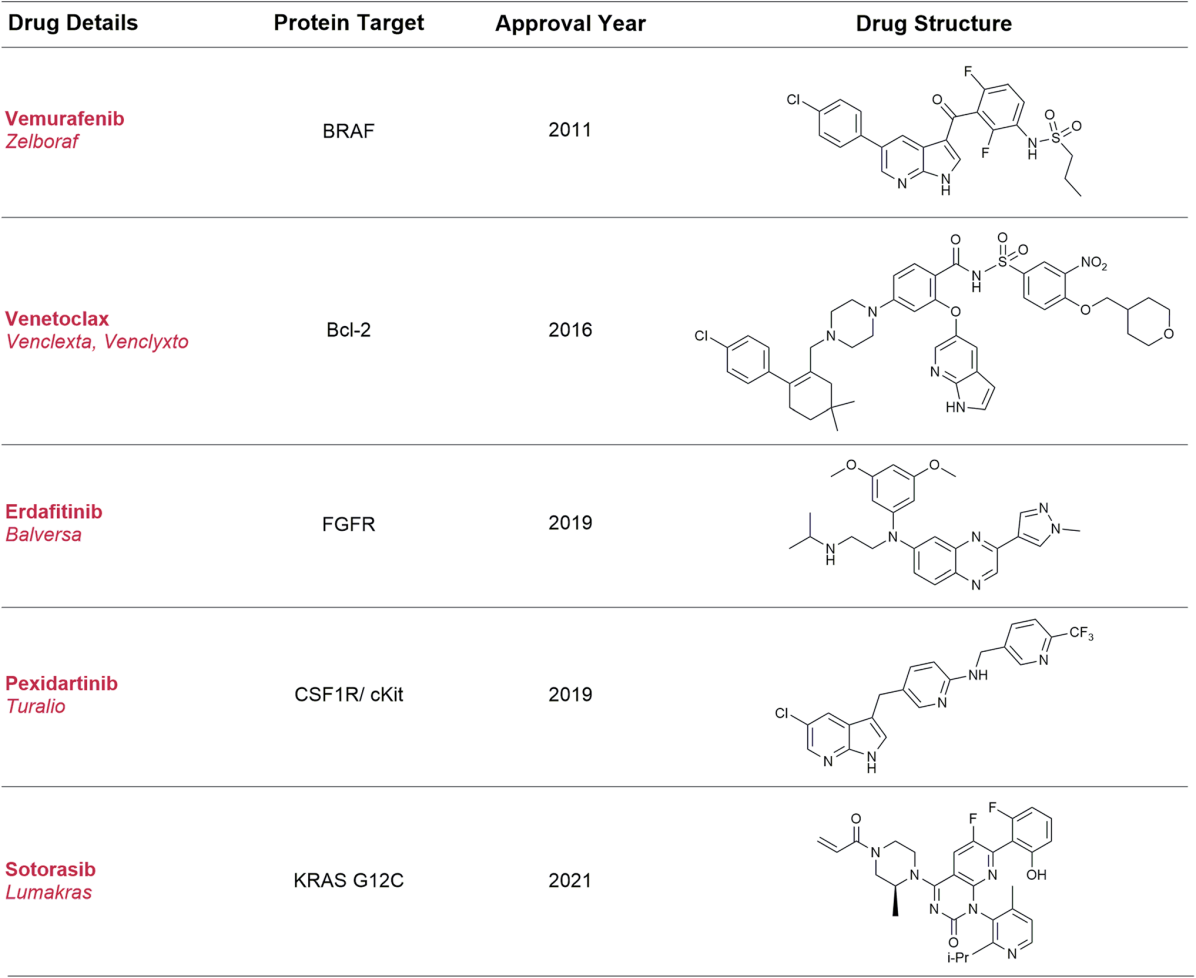
Approved drugs derived using the FBDD method. Sotorasib is an example of fragment screening using a covalent tethering approach and differs from the traditional examples in this table.

Broadly speaking, fragment-based drug discovery (FBDD) involves two steps: (1) the screening of a library of small, ligand-efficient§§Ligand efficiency is a parameter calculating the binding energy per heavy (non-hydrogen) atom of the ligand: LE = Δ*G*/*N*_non-hydrogen atoms_. organic molecules (fragments) against a biomolecule drug target of interest and (2) rational structure-guided design and optimisation of these fragments into bespoke molecules with improved target affinity, using X-ray crystallography and computational modelling. To date five FBDD-derived drugs (Fig. 1) have been approved, and global sales of Venetoclax alone were >US$ 1 billion in 2020.^10^

Although this approach to drug development is incredibly fruitful, it presents synthetic challenges over and above those seen with traditional medicinal chemistry approaches.^11^ Compared to hits identified through high-throughput screening (HTS) approaches, fragment hits are usually weaker and the fragment needs to be elaborated in a structure-guided fashion along specific points on the molecule (growth vectors) to pick up new interactions with the protein. This synthetic modification must occur in a manner which retains the key functionality (minimal pharmacophore)^12^ required for binding to the protein. Some fragments may have a good range of synthetic methods available to modify their growth vectors while others require resource-intensive experimentation to tailor literature conditions to the fragment of interest or the development of bespoke synthetic routes, thus delaying the drug discovery process.^11,13^

In our experience, we have encountered several in-house cases of fragment-to-lead (F2L) elaboration that have proven problematic as the protein architecture necessitated growth from C(sp^2^) and C(sp^3^) atoms originating on the fragment and these modifications were required in the presence of the fragment's polar functionality, which is required for binding.^13^ To examine how universal this challenge is to FBDD, we sought to investigate recent accounts of F2L campaigns reported in literature, and the findings of this analysis are reported herein. It is important to note that as this analysis is based on published examples of successful F2L programs, it could be skewed by a ‘survivorship bias’.^13^ The information regarding fragments that were not progressed, or particular fragment vectors not explored due to synthetic intractability, will not be captured in this dataset as they are not routinely reported or communicated to the FBDD-community. In view of this, we have disclosed an in-house case study where a fragment hit was not advanced due to synthetic challenges and used this to highlight the importance of continued development in organic synthesis (Section 4).

## 2. Constructing the dataset

A dataset of 131 FBDD examples highlighted in the five Mini-perspectives: *Fragment-to-Lead Medicinal Chemistry Publications* (2015–2019), was compiled.^14–18^ These FBDD campaigns covered a diverse range of target classes (24% kinases, 9% proteases, 36% other enzymes, 11% bromodomains, 14% protein–protein interactions and 6% other targets). The inclusion criteria for a fragment-to-lead campaign in these mini-perspectives were as follows:

• Fragment hit had a molecular weight (MW) <300 Da, consistent with the rule of three.¶¶Rule of three: in which fragments are defined as having a molecular weight <300, a cLogP ≤3, the number of hydrogen-bond donors is ≤3 and the number of hydrogen-bond acceptors is ≤3.^19^^19,20^

• Sources of fragment hits were screening (*e.g.* bioassay, biophysical method, X-ray, virtual screen, or any combination thereof), literature, or deconstruction of a known ligand.

• Potency/affinity of the lead is equal to or better than 2 μM.

• The improvement in potency/affinity from fragment to lead is at least 100-fold.

Throughout the majority of the F2L campaigns analysed in the dataset, the widespread use of X-ray or NMR derived structural information shows the core importance of structure-based drug design (SBDD) to FBDD. For the interest of the scientific community, we have provided a web-based viewer comprising this reported fragment, lead and protein X-ray structure information (where available) https://astx.com/interactive/F2L-2021/.^21^ This X-ray structure overlay page aids with viewing the polar interactions made by fragments to their target proteins and the vectors that are subsequently explored during F2L growth.

The assembled dataset was used to understand the different types of polar interactions required for fragment-protein binding, the atom types or groups grown from during F2L elaboration and the requisite bonds formed during this process. All this information is contained in Table S1 in the ESI.† In addition, examples taken from this table for use in figures are referred to by their relevant table entry number *e.g.*Fig. 3 2015-2 *etc.* By providing this analysis and the overlay page associated with it, we hope to inform the synthetic organic chemistry community of some of the specific synthetic challenges faced in FBDD and the scientific opportunities this presents to researchers.^3,11,13,22^

**Fig. 2:**
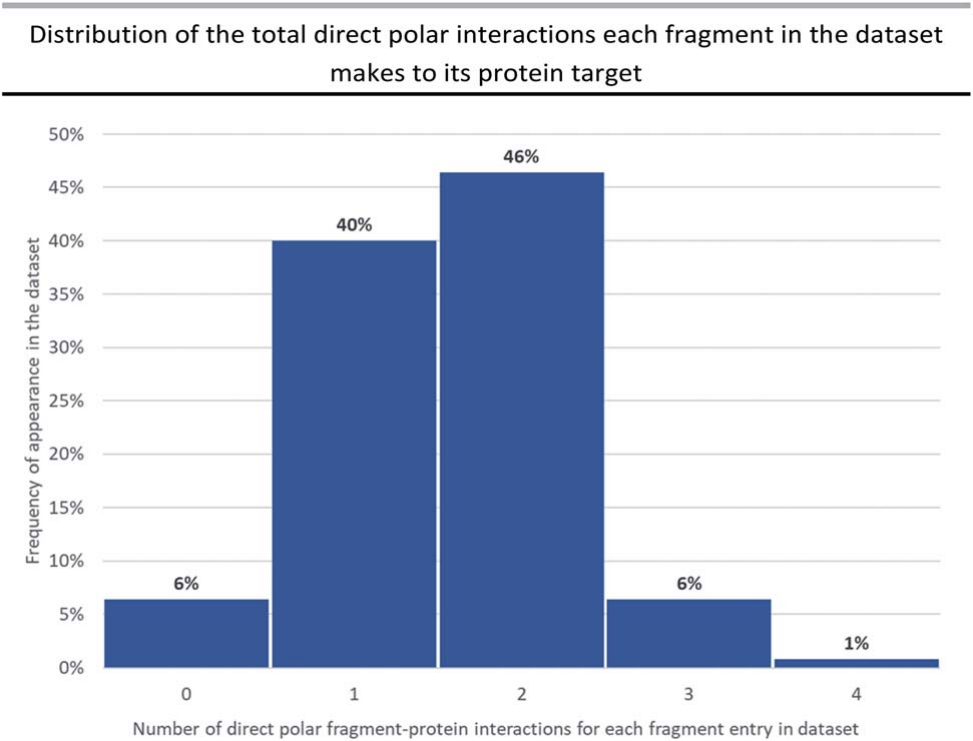
This chart shows the distribution of the total number of polar interactions each fragment entry in the dataset makes to its protein target, that are subsequently maintained in the lead. Fragments which make no interactions (bin 0, 6%) were those that were either exclusively lipophilic or were only making water-mediated polar interactions. 6 examples were excluded from the analysis either because their polar interactions were not conserved by the lead or because there was no structural or docking information available to define interactions.

**Fig. 3:**
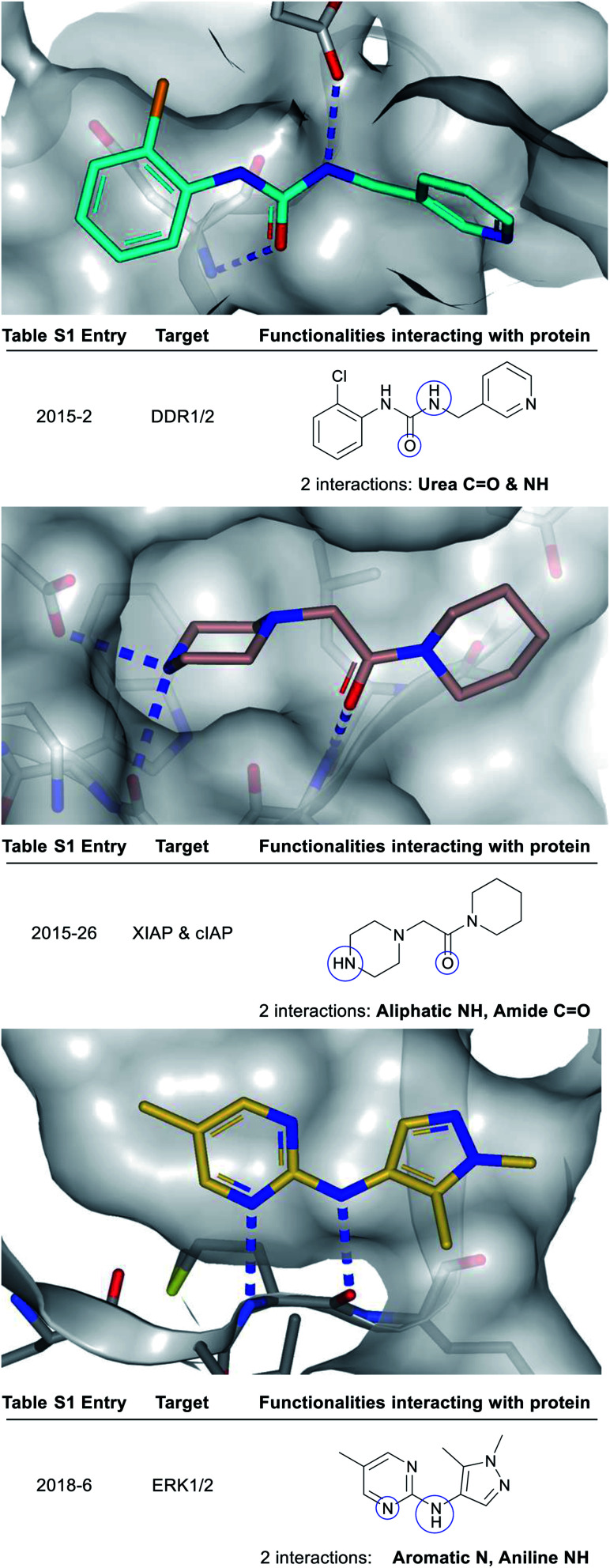
X-ray crystal structures showing polar groups on three example fragments selected from our analysis (see ESI Table S1† and web-based viewer^21^ entries as listed) involved in protein hydrogen-bonding interactions (blue dotted lines) and corresponding pictorial depiction of these interactions (blue circles). *PDB codes and references for these structures: 2015-2: 5bvk*,^24^*2015-26: 5c3h*,^25^*2018-6: 6g92*.^26^

### 2.1 Assignment of key polar fragment functionalities required for binding to proteins

Attractive electrostatic interactions between complementary hydrogen-bond donors/acceptors on the fragment and protein are often critical for initial fragment binding. Furthermore, they maintain the fragment–protein binding position during subsequent F2L elaboration and thus form a crucial part of the ‘minimal pharmacophore’ *i.e.* the minimum interactions required in fragment–protein binding.^12^

Previous analyses^23^ have documented all possible types of interactions that can form between a fragment and its protein target including: hydrogen-bonding direct to the protein or through water-mediated bridging contacts, arene-contacts (*i.e.* arene–arene stacking, arene–cation interactions *etc.*) and weaker interactions mediated by sulfur or halogens. To some extent, all fragment binding is driven by a degree of lipophilic character, however, for the purpose of our analysis we chose not to highlight these types of fragment–protein interactions and instead have focussed on polar interactions as these are highly directional and dominate the orientation of the growth vectors for a given fragment. The importance of polar interactions in FBDD is such that throughout our analysis we observed 93% of the dataset has at least one polar interaction between the fragment and protein that was subsequently conserved in the lead (Fig. 2).

Fig. 3 depicts a variety of polar binding groups which are designed into fragments to facilitate hydrogen-bonding with proteinogenic amino acids. These groups often contain hydrogen-bond donors, in the form of NH (from amines, anilines, azoles *etc.*) or OH (from alcohols *etc.*), or hydrogen-bond acceptors [*e.g.* ring N/O from aromatic heterocycles (azines, azoles *etc.*) or O from carbonyl (*e.g.* amides, ureas, ketones, *etc.*)]. The direct polar interactions observed between the fragment and protein (which are subsequently maintained in the lead) are highlighted as blue circles (Fig. 3, 5, 8 and ESI Fig. S1, S2 and Table S1†).

**Fig. 4:**
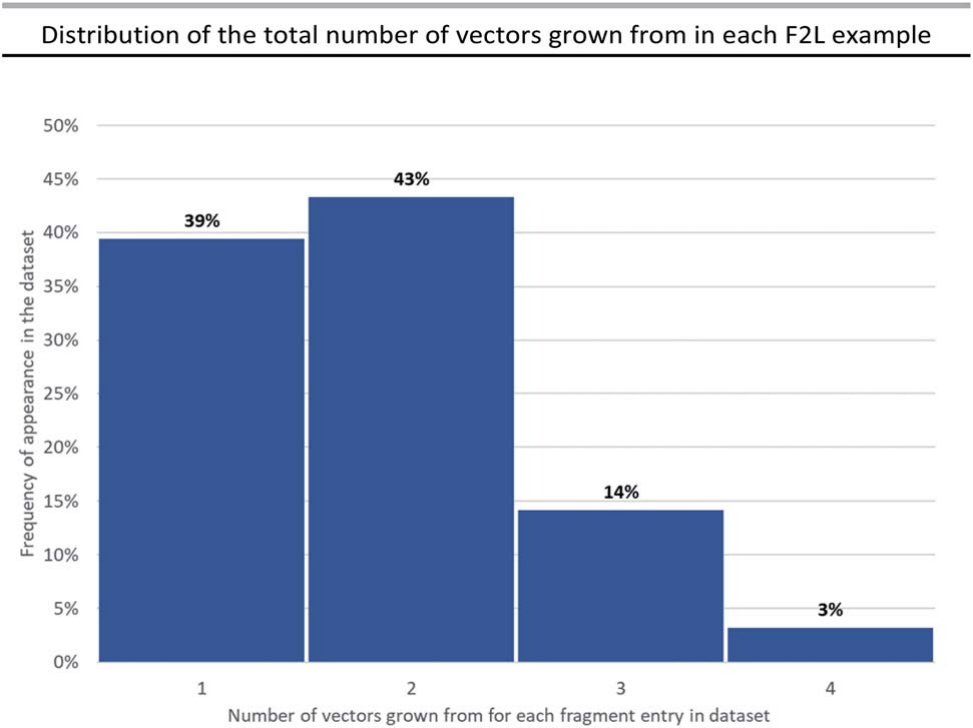
Distribution of the total number of vectors grown from in each F2L example in the dataset. Nominal vectors could not be assigned for 4 examples in the dataset due to presence of a scaffold hop from the original fragment.

**Fig. 5:**
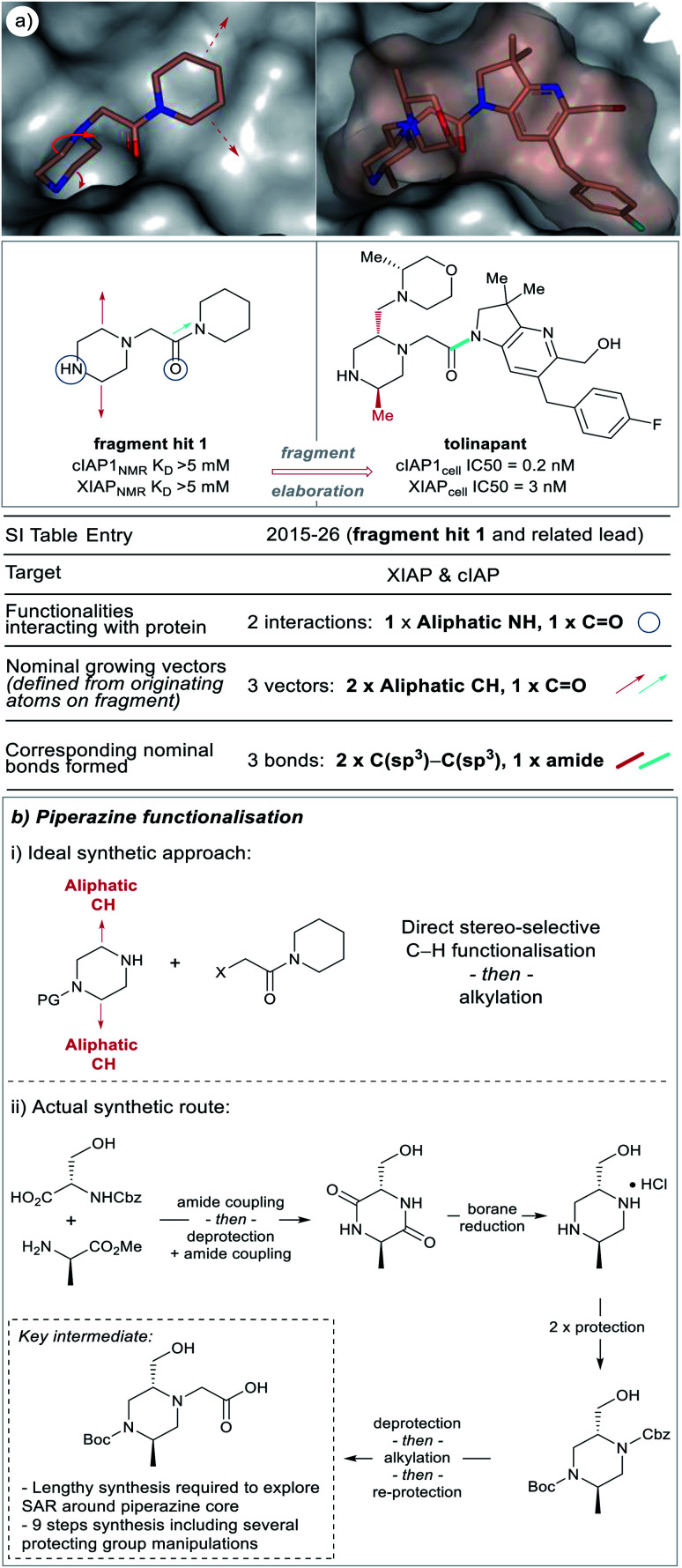
X-ray crystal structures of fragment **1** (*PDB code 5c3h*)^25^ and the clinical candidate (tolinapant) (*PDB code 5oqw*) binding to the target protein (XIAP) selected from our analysis (see ESI Table S1† and web-based viewer^21^ entries as listed for fragment hit **1** and a related lead compound).^27^ The polar binding groups on the fragment are identified (blue circles) in addition to the available growth vectors (red arrows on X-ray crystal structure). The new groups added onto the lead (red and cyan bonds, see: ‘Nominal growth’ and ‘Synthetically viable growth’ sections for more details) represent the observed bonds added to the fragment to generate the lead, these are nominal synthetic bonds, two arising from C–H positions on the fragment. Note that the easily modified secondary amine is not altered during the growth phase because this would disrupt the protein binding. Tolinapant is currently in phase 2 clinical trials.^39^

**Fig. 6:**
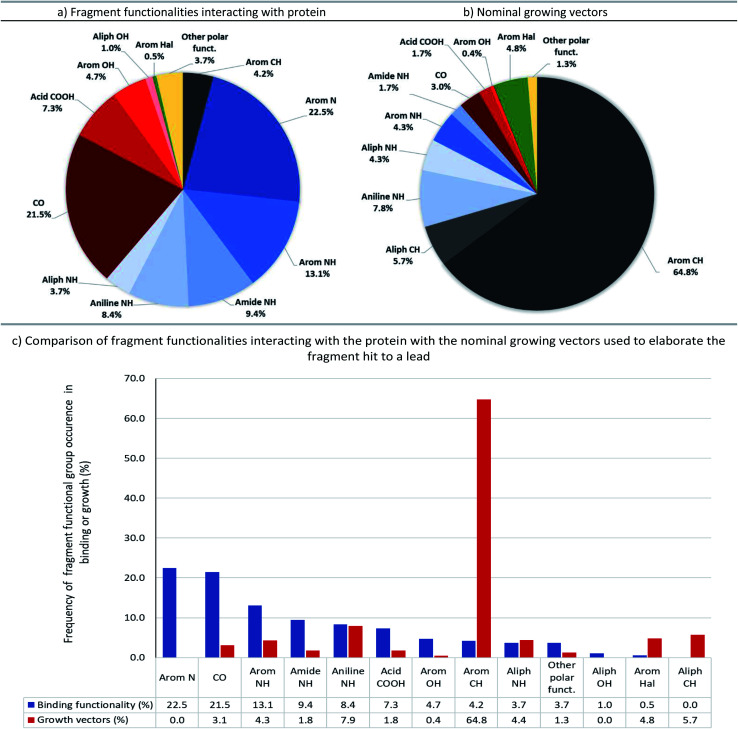
Pie-charts and bar-charts showing (a) fragment functionalities interacting with protein and (b) nominal growing vectors based on the 131 examples assessed. In the pie charts the segments are ordered C (black), N (blue), O (red), halogen (green), other (yellow) and within each family, *e.g.* nitrogen the segments are ordered by segment size. The groups most common in fragment binding are nitrogen (57%) and oxygen (35%) but in contrast the growing vectors are largely based on C–H vectors (71%).

**Fig. 7:**
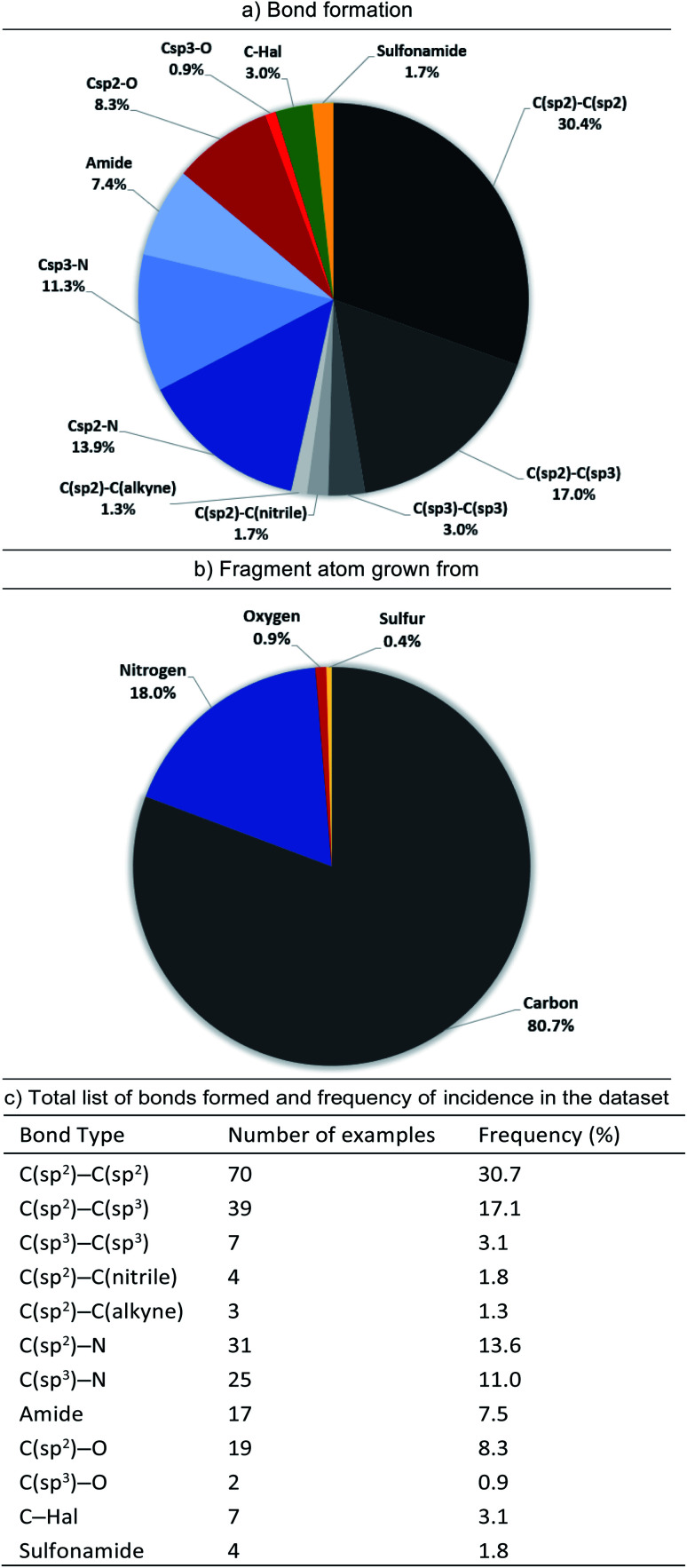
Pie-charts and table showing (a) the bond formation used in elaborating fragments to leads based on the 131 examples assessed (b) the specific fragment atom grown from and (c) the total list of bonds formed and their frequency in the dataset (bond formed is irrespective of the origin atom on fragment). In the pie-charts the segments are ordered C (black), N (blue), O (red), halogen (green), sulfur/other (yellow) and within each family *e.g.* nitrogen the segments are ordered by segment size. Pie-chart (a) shows the prevalence of C–C bond formation (54%) rather than C–heteroatom bonds (41%). Pie-chart (b) shows that ∼80% of growth is from a fragment carbon.

**Fig. 8:**
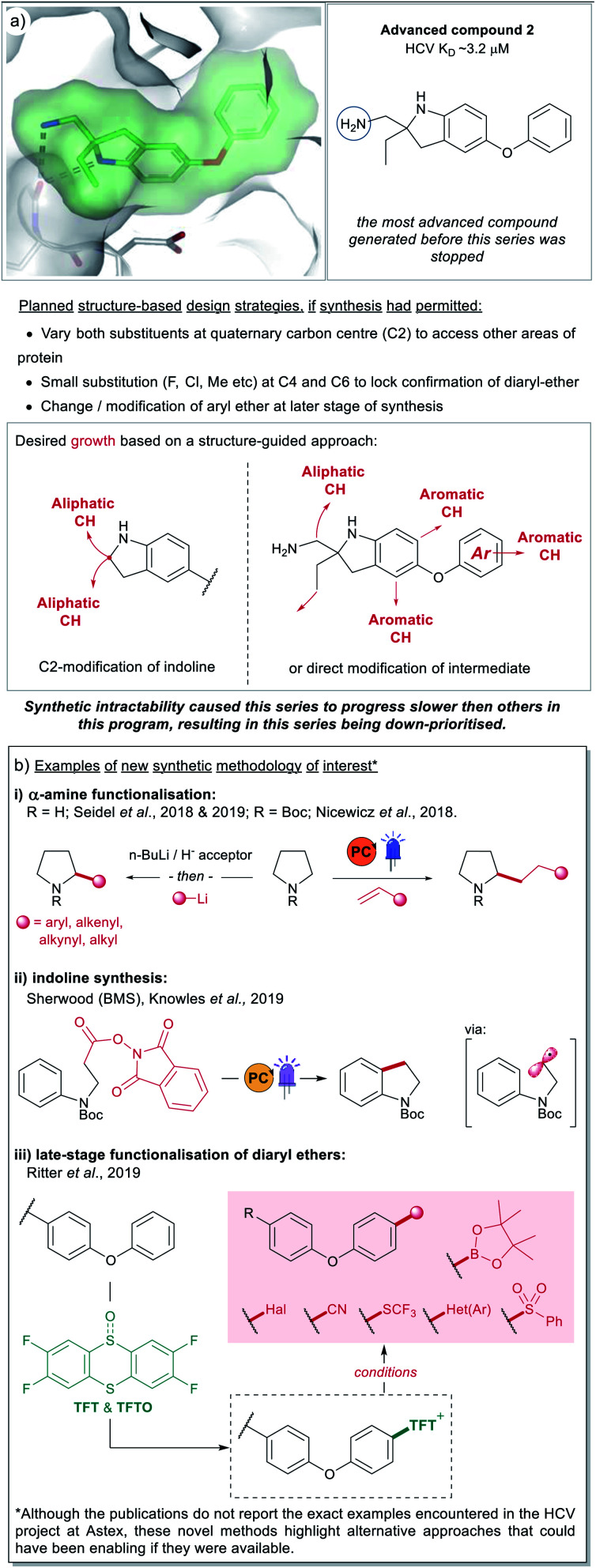
(a) An instance of a FBDD compound (**2**) where further elaboration was hampered due to synthetic tractability challenges. The areas of potential growth and associated design ideas are represented by the red arrows. The series progressed slowly due to synthetic challenges and was ultimately down-prioritised. (b) Examples of recently developed methodology (2017–2019) that could have enabled synthesis on the HCV project at Astex, if it had been accessible at the time.^28,35,36,61,62^

### 2.2 Assigning the atoms grown from, the nature of the groups added, and the bonds formed during fragment-to-lead (F2L) elaboration

During the F2L optimisation process, new groups are added to the fragment along well-defined growth vectors to increase protein-binding affinity (typically from mM to nM). This occurs through the formation of additional hydrogen bonds with the protein side chains/backbone and/or lipophilic/space-filling interactions with the 3D-architecture of the protein. The process of growth vector elaboration is used in FBDD and out of the F2L cases analysed in our dataset, the majority involved growth from 1 or 2 vectors with <20% of examples requiring modification from multiple vectors (growth from 3 vectors = 14% and growth from 4 vectors = 3%, Fig. 4). This highlights the ability of the FBDD process to grow a fragment into a lead in a precise, ligand-efficient manner along specific vectors guided by the protein structure; and, underlines the importance of synthetic methods that allow for selective functionalisation in the presence of polar functionalities.

Fig. 5a shows an example of a fragment hit (**1**) which was elaborated to generate a clinical candidate (tolinapant), affording *a* >10^6^-fold increase in potency for cIAP1 (*K*_D(fragment 1)_ = >5 mM (NMR) *vs.* cell IC_50(tolinapant)_ = 0.2 nM, Fig. 5).^25,27^ To understand: (1) what type of atoms the growth originates from, and (2) which types of groups are added to a fragment during F2L optimisation, we retrospectively defined the observed growth vectors that were used to evolve a fragment-hit into a lead throughout our dataset (Fig. 5 and 8 in manuscript and ESI Fig. S1, S2 and Table S1,† red and cyan bonds). These growth vectors represent the organic motifs that were added to the lead during this process. This classification was achieved by comparing the optimised lead structure against the starting fragment and highlighting the changes between the two. These highlighted bonds are therefore nominal and do not necessarily reflect the actual synthesis undertaken in the original publication, though in many cases it may be similar.

Most of the growth vectors in our analysis were defined by comparing the X-ray structures or dockings of the fragment and the lead. For those cases where no X-ray structural information was available, we have inferred growth vectors by comparison of the chemical structures of the hit and the lead. Our guidelines for assigning growth vectors are as follows (for further information see ESI†).

#### 2.2.1 Nominal growth (red bonds)

A growth vector is defined where a new group has been added to the fragment, when this is straightforward to delineate, a red bond is used to highlight this change (nominal growth vector).

In the F2L example shown in Fig. 5a, two alkyl groups are added to the piperazine portion of the fragment, these originate from aliphatic carbon-atoms (nominal growing vector = aliphatic CH) and are linked to the fragment through C(sp^3^)–C(sp^3^) bonds. An ideal scenario for an FBDD chemist working on this target would be the case shown through nominal growth (Fig. 5b(i)) where individual groups are appended to a core heterocycle (using C–H functionalisation), preferably in a stereo-defined manner. An approach like this would allow facile exploration of SAR through a convergent synthetic route. However, at the time, any incremental growth from the piperazine core required a lengthy *de novo* synthesis of the heterocyclic core from amino acids building blocks involving ∼9 steps and several protecting group manipulations (Fig. 5b(ii)).^25,27^ Recently, there have been several reports of synthetic methodology which can now permit direct C–H functionalisation of piperidine, piperazine and other aliphatic heterocycles and continued efforts in this area, particularly towards stereoselective methods are encouraged.^22,28–38^

#### 2.2.2 Synthetically viable growth (cyan bonds)

When comparing fragment and lead, if there is a synthetically straightforward alternative to direct growth from the nominal attachment points, the bond in question is deemed synthetically viable and such bonds are highlighted in cyan.

During our analysis, we found several examples of F2L growth that could not be defined by simply highlighting the bonds directly added to the fragment. In these cases, where growth required a core change or if modification of an analogous compound presented a more straightforward route, then a synthetically viable bond is instead highlighted in cyan (Fig. 5, ESI Table S1†). Fig. 5 shows one such instance where fragment growth to engage additional protein interactions required a core change: piperidine (fragment hit 1) → azaindoline (tolinapant). The available vectors (red dotted lines) can be clearly observed on the protein surface but, from the synthetic chemist's viewpoint, the vectors can be accessed much more easily by amide bond formation rather than using the nominal growing points. There are several examples of such synthetic opportunism and we have chosen to highlight these synthetically viable bonds, coloured cyan, and designated them as a growth vector for this analysis. Such a bond may be located in the core of the fragment as with fragment hit (**1**), (Fig. 5a, cyan C–N amide bond), and in this case corresponds to the bond formed by the FBDD chemists during the synthesis of this target.^25,27^ It is interesting to note that this fragment contains an embedded amide which could be perceived as a “poised fragment” even though it was not designed with this strategy in mind.^40^

## 3. Outcome of the analysis

The full output of our analysis can be found in the accompanying ESI (Table S1†) and is also available as a machine-readable csv file. This information details: (1) polar fragment functionalities interacting with the protein, (2) nominal growing vectors, and (3) the nominal bonds formed whilst growing the fragment into the lead. Fig. 6 summarises these data and compares the groups involved in fragment–protein interactions and those used as nominal growing vectors.

The most common polar functional groups involved in fragment–protein binding are: N–H hydrogen-bond donors (aromatic and aliphatic N–H, amides and anilines; totalling 35%), aromatic nitrogen hydrogen-bond acceptors (totalling 23%) and carbonyl oxygen group hydrogen-bond acceptors (on amides, ureas and ketones; totalling 22%). By contrast, only 18% of growing vectors originate from N–H groups and only 3% from carbonyl groups (primarily amides, ureas and ketones). Although amines can be readily elaborated through a variety of synthetic manipulations (alkylation: *via* S_N_2 attack on an electrophile or reductive amination; arylation: *via* S_N_Ar or transition-metal catalysed amination, *etc.*), this analysis shows that growth from amines (and polar groups in general) seldom occurs during F2L, probably because these moieties are often those making the key hydrogen-bonding interactions with the target protein (Fig. 6).

In contrast, C–H bonds are rarely encountered in polar fragment–protein binding interactions, due to their poor polarisability and small dipole moment (aromatic C–H totalling 4%, with no examples of aliphatic C–H). However, the majority of observed growing vectors originate from aromatic (65%) or aliphatic (6%) C–H's on the fragment (Fig. 6). Moreover, if we just consider the fragment atom elaborated during F2L, growth from carbon (in C–H, C–Hal, C=O groups *etc.*) accounts for 81% of the total cases analysed (Fig. 7).

When the types of bonds formed in nominal growth vector elaboration are compared (Fig. 7), a small proportion are what can be viewed as synthetically straightforward (C(sp^2^)–N = 14%, C(sp^3^)–N = 11%, C(sp^2^)–O = 8%, C(sp^3^)–O = 1%, amide = 8% and sulfonamide = 2%) and the majority of the total bonds formed are carbon–carbon bonds (54%). Accordingly, robust methods such as the Suzuki–Miyaura coupling are an invaluable tool in the medicinal chemist's arsenal for synthesising C(sp^2^)–C(sp^2^) bonds, however this requires access to the appropriately functionalised precursor molecules. However, hetero-aryl boronates are often unstable and can be challenging to synthesise,^41,42^ furthermore, small polar heterocycles can prove problematic in transition-metal catalysed cross-couplings by acting as ‘poisons’ resulting in catalyst deactivation.^43–46^

Certain C–C bonds can be quite challenging to synthesise in the presence of polar functionality. In the analysis we found a low incidence of C(sp^2^)–C(sp^3^) and C(sp^3^)–C(sp)^3^ bonds formed (17.1% and 3.1%, respectively and totalling 37% of all C–C bonds formed) furthermore, only 5.7% of the total growing vectors (or 8% of the total carbon-based growing vectors) originate from aliphatic C–H atoms. When these facts are considered it could be reasoned that the disproportionate occurrence of bonds formed with sp^3^-character^47^ in relation to sp^2^-character could be attributed to synthetic challenges that their inclusion presents. A recent Perspective by Caplin and Foley^48^ further emphasises the challenges associated with sp^3^-rich fragments as well as highlighting recent advances in C(sp^3^)–H functionalisation which are beginning to have an impact in this area.

## 4. Synthesis-biased *versus* structure-based design

In this section, we will discuss an example from a F2L project at Astex that was hampered by synthetic intractability. In 2012, Astex reported on an FBDD program targeting Hepatitis C virus NS3 protease-helicase.^49^ As part of this program previously unreported compound **2** was investigated (Fig. 8a). This compound was partially optimised from a much weaker fragment and analysis of the X-ray structure (Fig. 8a) indicated structure-based approaches for further affinity optimisation through subsequent growth (marked with red arrows). However, at the time this research was underway (prior to 2011), the synthesis of these design ideas was challenging for reasons that included:

• Constructing the quaternary centre at C2 to allow independent changes to both exocyclic substituents.

• Adding small substituents (F, Cl, Me) to C4 and C6 to lock the conformation of the diaryl ether.

• Incorporating changes to the terminal phenyl group at a late stage of the synthesis.

• The synthesis of **2** itself was time consuming, requiring 10 steps from *p*-amino diphenylether.

Given the challenges mentioned above and the time constraints typical for a drug discovery project, the partially optimised compound **2** was down-prioritised compared to other hits concurrently identified as binding to the same site on HCV.^13,49^ This is an example of the survivorship bias previously discussed and a potential contributing factor as to why C(sp^3^) vectors are of low incidence in our dataset.

Recently there have been several reports of new synthetic methodology which may address some of the aforementioned synthetic tractability issues, a select number are highlighted in Fig. 8b. These include methods for α-amine functionalisation which can allow installation of a variety of groups (alkyl, aryl, alkenyl and alkynyl) into cyclic amines (Fig. 8b(i)). We have chosen to highlight a small number of accounts from the groups of Nicewicz^28^ and Seidel,^35,36^ however there are numerous other reports of relevance in the literature.^32,50–60^ Although these publications do not include examples of di-functionalisation to form quaternary carbon centres, or the exact same indoline precursors, they give alternative options to a chemist trying to synthesise these types of architectures. As does the report from BMS and the Knowles group shown in Fig. 8b(ii),^61^ this work presents a novel route to indolines (and other semi-saturated bicyclic motifs) that would have presented welcome alternatives to the lengthy routes pursued by the chemists working on the HCV-project 10 years ago, at Astex.

Finally, we also wanted to showcase examples from the literature that could potentially have addressed the challenges around modifying the diarylether moeity in **2**. During the course of the project, the synthetic routes explored by the chemists started from *p*-amino diphenylether, with this aryl-ether motif being carried throughout the synthesis. At the time there were no available options for growth *via* late-stage modification and this meant that any SAR exploration around the phenyl-group required a lengthy *de novo* synthesis. Routes to access sterically hindered ethers worth noting include, metal-free iodonium salt-mediated arylation of phenols,^63,64^ and photocatalytic and electrochemical-mediated routes to alkyl ethers.^65,66^ Of particular interest is the late-stage C–H thianthrenation chemistry reported by Ritter *et al.* (Fig. 8b(iii)).^62^ This approach is particularly attractive as it allows the generation of a stable activated intermediate that is a competent functional handle in a variety of subsequent transformations. Furthermore, the initial pre-activation step can be performed in the presence of several different un-protected polar functionality and Lewis-basic heterocycles and will likely see good uptake within industrial settings.

## 5. Conclusions and take-home message

FBDD is practised in academic, pharma and biotech laboratories and to date, has led to 5 launched drugs. One of the main scientific challenges in FBDD is the carefully designed and executed elaboration of a fragment into a lead in the presence of the fragment's polar binding functionality.

Heteroatom-mediated polar interactions play an important role in molecular recognition of the fragment by the protein, with 93% of the examples in our analysis making at least one hydrogen-bond to their target protein (Fig. 2). Moreover, there is high conservation of these hydrogen-bonds on growing to a lead which not only shows the fundamental importance of polar interactions in FBDD but that these hetero-atom rich groups are not viable points for synthetic growth.

Our analysis has shown that the majority (∼80%) of fragment growth originates on carbon atoms and, furthermore, that ∼54% of the bonds being formed are C–C (Fig. 7). Thus, continued development of C–C bond formations with high functional group compatibility will be of high value for the FBDD community. Of particular use would be mild, site-selective C–H functionalisation on heteroaromatics (HCV example Fig. 8), positional C(sp^3^)–H functionalisation to form tertiary (IAP example Fig. 5) and quaternary (HCV example Fig. 8) stereocentres. In the case of the latter examples, these synthetic challenges are likely a contributing factor to the low incidence of C(sp^3^) bond formations seen in our analysis.

An “ideal synthesis”^67^ of a lead would allow: (1) site-selective formation of bonds at all growing points of a fragment, (2) whilst being mild enough to be compatible with essential polar functionality, and (3) proceeding with minimal or no need for protecting groups. Such synthetic advances which enable facile routes to structure-based target-molecules, without extensive experimentation or long, protecting group-heavy syntheses would both speed up the F2L design cycle as well as potentially prevent fragment series from being abandoned due to synthetic intractability.

We believe that further development of C–H functionalisation that is tolerant to polar fragments has the potential to transform FBDD.

## Data availability

The datasets supporting this article have been uploaded as part of the ESI†

## Author contributions

G. C. and D. C. R. conceived the idea for this analysis. G. C., R. G., R. S. H., P. N. M. and D. C. R. performed the analysis. F. L. and P. N. M. prepared and made available the online overlay page. R. G. and R. S. H. wrote the manuscript.

## Conflicts of interest

The authors are employees of Astex Pharmaceuticals.

## Supplementary Material

SC-012-D1SC03563K-s001

SC-012-D1SC03563K-s002

SC-012-D1SC03563K-s003
