# Supplementary material for: C–H functionalisation tolerant to polar groups could transform fragment-based drug discovery (FBDD)
Source: Chem Sci. 2021 Sep 1;12(36):11976–85. doi: 10.1039/d1sc03563k (PMC8457390; doi:10.1039/d1sc03563k)
Supplement: SC-012-D1SC03563K-s001 [file SC-012-D1SC03563K-s001.pdf]

Electronic Supplementary Material (ESI) for Chemical Science.
This journal is © The Royal Society of Chemistry 2021

C-H functionalisation tolerant to polar groups could transform fragment-based drug discovery (FBDD)

Gianni Chessari, Rachel Grainger,* Rhian S. Holvey,* R. Frederick Ludlow, Paul N. Mortenson and David C. Rees

Assignment of key polar fragment functionalities required for binding to protein

Using the 131 examples of FBDD campaigns detailed in the five Mini-perspectives: Fragment-to-Lead Medicinal Chemistry Publications (2015-2019),[1-5] we initially examined the X-ray or NMR structural information of both the hit and lead (where available) to define the types of fragment polar functional groups making direct interactions with proteinogenic amino acid groups. Water mediated and/or interactions that were not maintained by the lead compound were discounted, as instead we chose to only focus on key hydrogen-bonding interactions required for fragment-protein binding (for further discussion see main text of manuscript).

Pleasingly, the majority (96/131; 73%) of the hit-to-lead papers analysed in this published dataset, had X-ray or NMR structural information detailing the binding of both the fragment hit and lead (or close analogues thereof) to the protein of interest. In some cases, however, structural information for either the hit, lead or both was missing but a putative binding mode was suggested through computational modelling (23/131; 18%), we have highlighted these cases accordingly (Footnote 1, Table S1). For a small number of examples (10/131; 8%), no structural information was available for either the hit, lead or both, therefore determination of the key fragment polar functionalities interacting with the protein was not possible (entries listed in Footnote 2, Table S2). Furthermore, in some cases there was a shift in binding mode for the lead compared to the fragment (13/131; 10%) or the core structure of the fragment was changed enough that it was perceived to be a scaffold hop (6/131; 5%). We have highlighted these examples in our analysis and only assigned fragment polar functionalities interacting with the protein that were maintained by the lead.

Defining growth vectors

We recognise that defining nominal growth vectors is somewhat subjective, so we created a set of guidelines to try to ensure consistency (Supplementary Information, Figure S1).

- Nominal growth vectors are highlighted as red bonds, when it is not synthetically sensible to highlight the observed change as nominal growth, a synthetically viable bond is instead highlighted in cyan, (e.g. Figure S2, 2015-17)
- A growth vector is defined as being where a new group has been added to the fragment, even if this group is small e.g. ArC–H → ArC–Me (Figure S1, 2015-2)
- If a pre-existing group is modified only slightly (e.g. homologation/ dehomologation) and does not engage any additional protein interactions, this is not counted as a growth vector e.g. nPr → Et (Figure S1, 2015-6)
- If a ring or heterocycle has been changed or expanded, without changing the pharmacophore, this is not defined e.g. pyridine → pyrazole (Figure S1, 2015-7), 6- → 7-membered ring expansion (Figure S1, 2015-4)
- Groups removed from a fragment are not highlighted e.g. ArC–Cl → ArC–H (Figure S1, 2015-2)
- In some cases, a fragment atom was changed to enable a growth vector, this has been highlighted e.g. pyridyl-N → phenyl-CH (Figure S1, 2015-2)
- If a heteroatom has been added to the initial fragment scaffold, this is highlighted in red even if this is not a growth vector (Figure S1, 2019-1), we have done this to highlight the breadth of different heterocycles encountered in FBDD
- The type of bond being formed when growing from the fragment is defined irrespective of the starting fragment atom e.g. the C(sp^2)–N segment includes cases where a nitrogen is added to a fragment-C(sp^2) atom and where a C(sp^2) atom (e.g. arene or alkene) is added to a nitrogen atom located on the fragment

For the majority of the cases in Table S1, defining nominal growth vectors under the constraints listed above was relatively straightforward, however, some cases were more challenging and Figure S2 details a number of select examples to illustrate the range of situations encountered during this analysis. For example, in entry 2015-1 (Figure S2), the fragment hit is entirely encompassed by the lead and one ArC–H has been elaborated with a C–C coupling,

this case is clear-cut. Conversely, entry 2015-17 (Figure S2) shows an example where the approximate designation of growth clearly conflicted with what was synthetically viable. Here, nominal growth is observed to be double alkylation of the amide N–H (shown with red arrows), however amide bond formation is synthetically straightforward and would permit a greater scope of analogues accessible in SAR exploration. In instances like this, the synthetically viable, rather than the strictly nominal, growth vector has been defined (Table S1 & Figure S2, cyan bonds).

In our analysis, we also found examples requiring both the designation of a strictly nominal (red bond) and a more synthetically viable growth vector (cyan bond). This is highlighted in the case of 2017-14 (Figure S2), where ArC–F → to ArC–OAr growth is nominal (red bond), however, the nominal growth vector of the sulfonamide is observed to be from the CH of the methyl group. Considering the robustness of sulfonamide chemistry and the challenge of methyl C–H activation, we have defined the synthetically viable bond between the aniline and the sulfur as being the growth for this case (Figure S2, cyan bond).

We have also encountered more complex examples when defining growth vectors in this dataset, such as 2019-16 (Figure S2). In this example, though the change of an aromatic ethyl to a phenyl can be defined as a simple nominal growth vector, designating the other vectors proved more difficult due to inverted stereochemistry between the fragment and the lead, in addition to the change in linking atom within the fragment scaffold. In this case, we have defined the ArC–N → ArC–O as a synthetically viable growth vector but have also highlighted the methyl → benzyl switch at the stereogenic centre as this comprises both the nominal growth and a change in stereochemistry from the initial fragment (Figure S2).

Table Entry	Fragment	Lead	Guidelines for defining vectors and growth (specific examples higlighted in lead)
2015-2	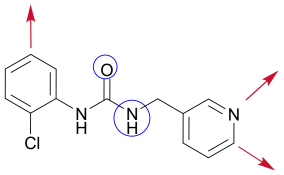	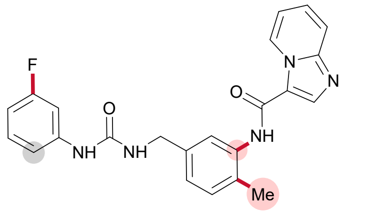	**C_{Ar}–Cl → C_{Ar}–H** *not defined* Groups removed from the original fragment during elaboration are not highlighted <br>**N → C (pyridine to benzene)** *defined* Atom changed in fragment to enable synthetic growth <br>**C_{Ar}–H → C_{Ar}–Me** *defined* Growth highlighted even if the group added is small
2015-6	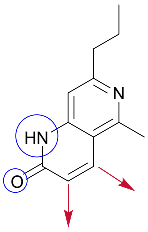	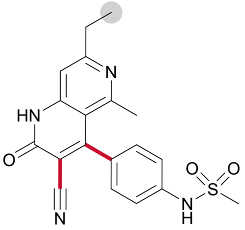	**C_{Ar}–H → C_{Ar}–Me** *not defined* Modification of pre-existing group on fragment does not engage additional protein interactions ∴ not defined as a growth vector
2015-4	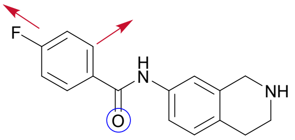	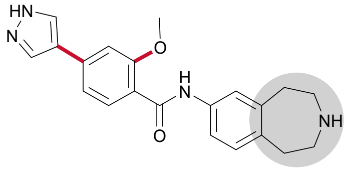	**6 → 7 membered cyclic amine** *not defined* Ring expanded to improve solvent interactions and not required to enable growth or synthesis
2015-7	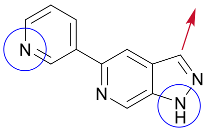	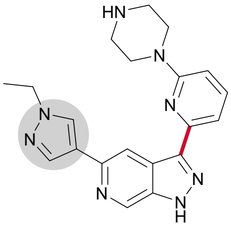	**Pyridine → Pyrazole** *not defined* Heterocycle changed but pharmacophore is maintained and no additional protein interactions are acquired
2019-1	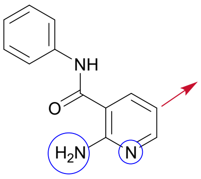	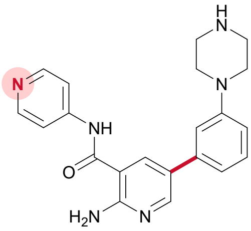	**C → N (benzene to pyridine)** *highlighted* Heteroatom added during elaboration to pick up additional protein interactions

Key:

- Fragment functionality required for protein binding
- Growth vector (origin and direction of growth during fragment to lead elaboration)
- Group highlighted to show where growth was defined
- Group highlighted to explain why growth was not defined
- Bond formed during growth vector elaboration (nominal / observed growth)

Figure S1 Illustrates the guidelines we used to define nominal growth vectors. Fragment and corresponding lead showing the fragment polar binding groups (blue circles) and the nominal fragment growth vectors (red arrows). The new binding groups added onto the lead during fragment elaboration represent hypothetical synthetic bonds (red or cyan bonds). Guidelines for defining growth vectors are summarised in the final column.

Table Entry	Fragment	Lead	Growth Vectors	Bond Designation
2015-1	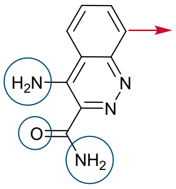	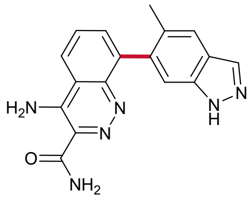	1 growth vector (C_{Ar}–H)	Assigned growth (red): **C_{Ar}–H → C_{Ar}–C_{Ar}**
2015-17	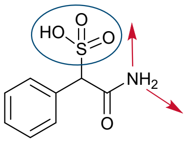	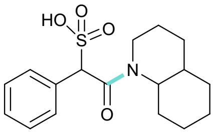	2 growth vectors (2 x NH)	Assigned growth (cyan): **amide bond†**
2017-14	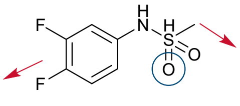	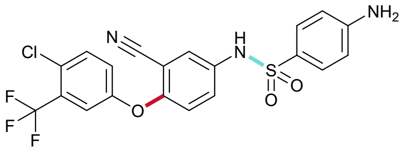	2 growth vectors (C_{Ar}–F & C_{Alkyl}–H)	Assigned growth (red): **C_{Ar}–F → C_{Ar}–OAr** Assigned growth (cyan): **sulfonamide bond†**
2019-16	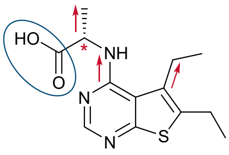	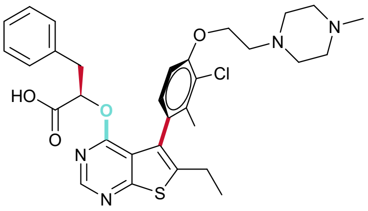	3 growth vectors (2 x C_{Ar}–H & *C_{Alkyl}–H) *n.b. includes change in stereochemistry*	Assigned growth (red): ***C_{alkyl}–H → *C_{alkyl}–C_{alkyl}** Assigned growth (red): **C_{Ar}–H → C_{Ar}–C_{Ar}** Assigned growth (cyan): **aryl ether†**

Key:

Fragment functionality required for protein binding

Growth vector (origin and direction of growth during fragment to lead elaboration)

Bond formed during growth vector elaboration (nominal / observed growth)

Synthetically viable bond defined (in the absence of pertinant nominal growth)

† Defined bond is more synthetically viable to enable scope

Figure S2 Shows specific examples of nominal and or synthetically viable growth. For each entry, the polar binding groups on the fragment are highlighted (blue circles) in addition to the nominal fragment growth vectors elaborated in the lead to increase binding affinity (red arrows). The new binding groups added onto the lead during fragment elaboration represent hypothetical synthetic bonds (red or cyan bonds).

Astex Overlay Page Help https://astx.com/interactive/F2L-2021/

Overview

The overlay pages provide a curated view of a series of protein-ligand structures. The structures can be explored and displayed through the heirarchical menus in the right hand panel.

Structures have some basic top-level controls: checkboxes and colour pickers to control the protein, ligand, waters and simple molecular surfaces.

Expanding a structure displays further controls for different display styles and controls to turn on electron density maps (where available). The maps are often clipped to the immediate vicinity of the ligand to minimize file sizes.

Mouse Controls

- **Rotate** - *Left* button hold and move
- **Zoom** - *Shift+Left* button hold and move, **OR** *Right* button hold and drag (up/down)
- **Translate** - *Ctrl+Left* button hold and move
- **Adjust clipping planes** - *Scroll* mousewheel (**OR** "-" and "+" keys)
- **Pick** - *Left* click on an atom (see measurements below)
- **Centre** -*Middle* click on an atom or bond

Keyboard Shortcuts

The following keyboard shortcuts are available when the NGL Viewer has focus (i.e. after you click on the viewer area).

General

- (***c***)entre – recentre on the last picked atom
- (***r***)eset – zooms to view all *loaded* structures
- Sp(***i***)n – toggle spin mode
- Roc(***k***) – toggle rock mode
- (_-_) – decrease depth-of-field (move clipping planes together) **OR** mouse scrollwheel up
- (_+_) – increase depth of field (move clipping planes apart) **OR** mouse scrollwheel down

Measurements
Pick to select atoms, then:

- (***d***)istance – operates on last two picked atoms
- (***a***)ngle – operates on last three picked atoms
- (***t***)orsion – operates on last four picked atoms

(***Shift-d/a/t***) clears distances, angles, torsions respectively

References

1. C. N. Johnson, D. A. Erlanson, C. W. Murray and D. C. Rees, *Journal of Medicinal Chemistry*, 2017, **60**, 89-99.
2. C. N. Johnson, D. A. Erlanson, W. Jahnke, P. N. Mortenson and D. C. Rees, *Journal of Medicinal Chemistry*, 2018, **61**, 1774-1784.
3. P. N. Mortenson, D. A. Erlanson, I. J. P. de Esch, W. Jahnke and C. N. Johnson, *Journal of Medicinal Chemistry*, 2019, **62**, 3857-3872.
4. D. A. Erlanson, I. J. P. de Esch, W. Jahnke, C. N. Johnson and P. N. Mortenson, *Journal of Medicinal Chemistry*, 2020, **63**, 4430-4444.
5. W. Jahnke, D. A. Erlanson, I. J. P. de Esch, C. N. Johnson, P. N. Mortenson, Y. Ochi and T. Urushima, *Journal of Medicinal Chemistry*, 2020, **63**, 15494-15507.

Total entries		131																	191															230												230		
									8	0	43	25	16	7	18	41	14	9	2	1	7		149	13	0	10	18	10	4	7	4	1	0	11	3	70	39	7	3	4	32	26	17	19	2	7	4
								Fragment functionalities interacting with proteins														Nominal growing vectors														Bond formation											
Year	Entry	Fragment Hit + PDB Code (where available)	Lead + PDB Code (where available)	Institution	Target	Binding pose changed?	Scaffold Hop?	# fragment / protein interactions	Arom CH	Aliph CH	Arom N	Arom NH	Aniline NH	Aliph NH	Amide NH	CO	Acid COOH	Arom OH	Aliph OH	Arom Hal	Other polar funct.	# nominal growing vectors	Arom CH	Aliph CH	Arom N	Arom NH	Aniline NH	Aliph NH	Amide NH	CO	Acid COOH	Arom OH	Aliph OH	Arom Hal	Other polar funct.	C(sp2)-C(sp2)	C(sp2)-C(sp3)	C(sp3)-C(sp3)	C(sp2)-C(alkyne)	C(sp2)-C(nitrile)	Csp2-N	Csp3-N	amide	Csp2-O	Csp3-O	C-Hal	sulfonamide
2015	1	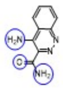 4ZLY	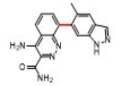 4Z3V	Takeda	BTK	N	N	3					1		1	1						1	1													1											
2015	2	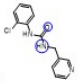 5BVK	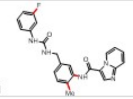 5BVN	Astex	DDR1/2	N	N	2							1	1						3	3														1				1					1	
2015	3	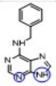 4QP1	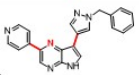 4QPA	Genentech	ERK2	Y	N	2			1	1										2	2													2											
2015	4	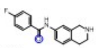 4UMR	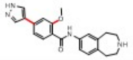 4D2V	Astex, Janssen	MELK	N	N	1								1						2	2													1								1			
2015	5	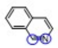	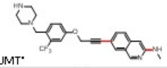 4UMT*	Astex, Janssen	MELK	N	N	2	1		1											2	2																1		1						
2015	6	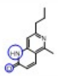 4UNP	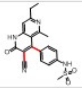 4UNS	AZ	Mtb TMK	N	N	2				1				1						2	2													1				1							
2015	7	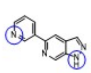 5DHJ	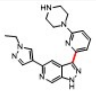 5DIA*	Genentech	Pim 2K	N	N	2			1	1										1	1													1											
2015	8	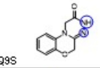 4Q9S	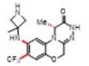 4Q9Z	Abbvie	PKC-theta	N	N	2			1				1							3	2	1													1	1			1						
2015	9	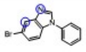	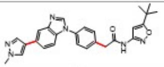	Naple, Arizona & Synactix	RET, VEGFR2	N	N	2	1		1											2	1											1		1	1										
2015	10	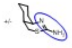 4ZSM	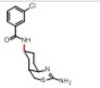 4ZSP	Lilly	BACE1	N	N	1													1	1		1																		1					
2015	11	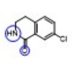 4X8T	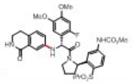 4X8V*	BMS	Factor VIIa	N	N	2							1	1						1												1							1						
2015	12	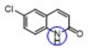 4CR5	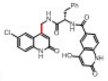 4CRF	AZ	FXIa	N	N	1				1										1	1														1										
2015	13	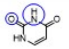	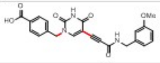	Zurich	MMP-13	N	N	2							1	1						2	1						1										1			1					
2015	14	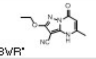 5BWR*	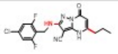 5BWX*	GSK, Strathclyde	BCATm	N	N															2	2														1				1						

Table S1 An assessment of 131 Fragment-to-Lead campaigns detailing i) polar fragment functionality interacting with proteins, ii) the nature of the atom growth originated from during fragment-to- lead elaboration and iii) the observed bonds formed during this process.

								191													
Total entries		131							8	0	43	25	16	7	18	41	14	9	2	1	7
								Fragment functionalities interacting with proteins													
Year	Entry	Fragment Hit + PDB Code (where available)	Lead + PDB Code (where available)	Institution	Target	Binding pose changed?	Scaffold Hop?	# fragment / protein interactions	Arom CH	Aliph CH	Arom N	Arom NH	Aniline NH	Aliph NH	Amide NH	CO	Acid COOH	Arom OH	Aliph OH	Arom Hal	Other polar funct.
2015	15	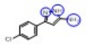 5BOD*	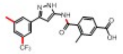	A*STAR	DNA gyrase B	N	N	3			1	1	1								
2015	16	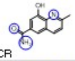 4UCR	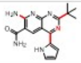 4UCO	AZ	LigA	N	N	3			1				1	1					
2015	17	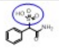	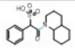	Indiana	mPTPB	N	N	1													1
2015	18	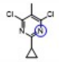 5C1W	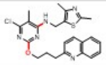 5C2H	Merck	Phopshodiesterase 10A	N	N	1			1										
2015	19	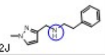 4Y2J	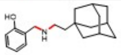 4Y2X	Astellas	Soluble epoxide hydrolase	N	N	1						1							
2015	20	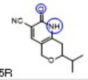 5C5R	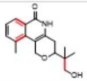 5C5Q*	Roche	Tankyrase	N	N	2				1				1					
2015	21	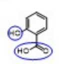	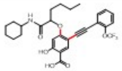	Indiana, Texas, UCSD	UBLCP1	N	N	2									1	1			
2015	22	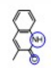 5A5O	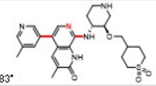 5A83*	GSK	ATAD2	N	N	2				1				1					
2015	23	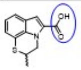	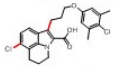 4ZBI	Vanderbilt	Mcl-1	N	N	1									1				
2015	24	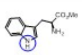 4B2L	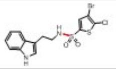	Cambridge	RAD51		N	1				1									
2015	25	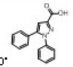 4LUO*	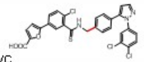 4LWC	Vanderbilt	RPA70N	Y	N														
2015	26	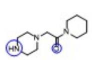 5C3H	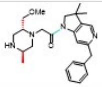 5C84*	Astex	XIAP & cIAP	N	N	2						1		1					
2015	27	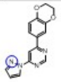	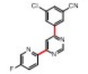 5CGD	Heptares	mGluR5	N	N	1			1										
2016	1	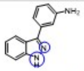	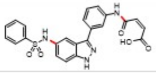	National Tsung Hua	Aurora A/B	N	N	2			1	1									
2016	2	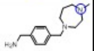 5EQE	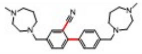 5EQY	ARIAD	Choline Kinase α	N	N	1						1							

		230													
			149	13	0	10	18	10	4	7	4	1	0	11	3
		Nominal growing vectors													
Year	Entry	# nominal growing vectors	Arom CH	Aliph CH	Arom N	Arom NH	Aniline NH	Aliph NH	Amide NH	CO	Acid COOH	Arom OH	Aliph OH	Arom Hal	Other polar funct.
2015	15	3	2				1								
2015	16	3	3												
2015	17	1								1					
2015	18	2	1											1	
2015	19	2						2							
2015	20	3	2	1											
2015	21	2	2												
2015	22	2	2												
2015	23	2	2												
2015	24	1						1							
2015	25	1	1												
2015	26	3		2						1					
2015	27	2	2												
2016	1	2	1				1								
2016	2	2	2												

		230											
		70	39	7	3	4	32	26	17	19	2	7	4
		Bond formation											
Year	Entry	C(sp2)-C(sp2)	C(sp2)-C(sp3)	C(sp3)-C(sp3)	C(sp2)-C(alkyne)	C(sp2)-C(nitrile)	Csp2-N	Csp3-N	amide	Csp2-O	Csp3-O	C-Hal	sulfonamide
2015	15		2						1				
2015	16	1	1				1						
2015	17								1				
2015	18						1			1			
2015	19							2					
2015	20	2		1									
2015	21				1					1			
2015	22	1					1						
2015	23		1									1	
2015	24												1
2015	25		1										
2015	26			2					1				
2015	27	2											
2016	1						1		1				
2016	2	1				1							

Table S1 An assessment of 131 Fragment-to-Lead campaigns detailing i) polar fragment functionality interacting with proteins, ii) the nature of the atom growth originated from during fragment-to- lead elaboration and iii) the observed bonds formed during this process.

Total entries			131				
**Year**	**Entry**	**Fragment Hit + PDB Code (where available)**	**Lead + PDB Code (where available)**	**Institution**	**Target**	**Binding pose changed?**	**Scaffold Hop?**
2016	3	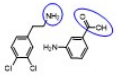 5CLP & 5CSV	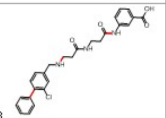 5CU3	Cambridge	CK2α	N	N
2016	4	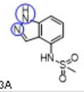 5L3A	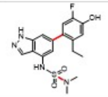	LEO Pharma	JAK1, JAK2, JAK3		N
2016	5	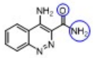	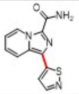	Takeda	MKK3/6	N	N
2016	6	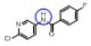	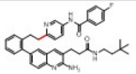 5I3Y	Amgen	BACE1	N	N
2016	7	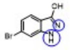	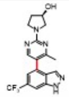 5JI6*	Takeda	MetAP2	N	N
2016	8	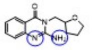	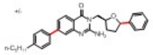 4Z22*	Latvian institute of Org Syn and Crick	Plasmepsin II	N	N
2016	9	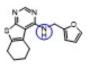 5SY3	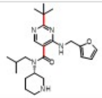 5KOQ	Takeda	Renin	Y	N
2016	10	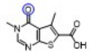 5I5V	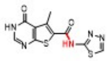 5I5X	GSK, Strathclyde	BCATm	N	N
2016	11	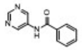 5I5W	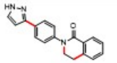 5I60*	GSK, Strathclyde	BCATm	N	N
2016	12	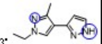 5K03*	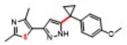 5K0L	Roche	Catechol O-methyltransferase	N	N
2016	13	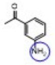 3R59	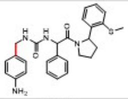 4J5C*	Univ Paris Est, Univ Montpellier	Cyclophillin D	N	N
2016	14	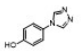 4G47	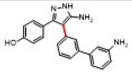 5IBE	Cambridge, Manchester, Sao Paolo, Crick	CYP121	Y	N
2016	15	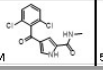 5DTM	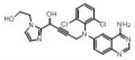 5DTR*	Novartis	DOT1L	N	Y

			191												
			8	0	43	25	16	7	18	41	14	9	2	1	7
			Fragment functionalities interacting with proteins												
**Year**	**Entry**	# fragment / protein interactions	Arom CH	Aliph CH	Arom N	Arom NH	Aniline NH	Aliph NH	Amide NH	CO	Acid COOH	Arom OH	Aliph OH	Arom Hal	Other polar funct.
2016	3	2						1			1				
2016	4	2			1	1									
2016	5	2							1	1					
2016	6	1							1						
2016	7	2			1	1									
2016	8	2			1		1								
2016	9	1						1							
2016	10	1								1					
2016	11														
2016	12	2			1	1									
2016	13	1					1								
2016	14	1													
2016	15														

			230												
			149	13	0	10	18	10	4	7	4	1	0	11	3
			Nominal growing vectors												
**Year**	**Entry**	# nominal growing vectors	Arom CH	Aliph CH	Arom N	Arom NH	Aniline NH	Aliph NH	Amide NH	CO	Acid COOH	Arom OH	Aliph OH	Arom Hal	Other polar funct.
2016	3	3					1	1						1	
2016	4	2	1												1
2016	5	1	1												
2016	6	1												1	
2016	7	1	1												
2016	8	2	1	1											
2016	9	2	2												
2016	10	1									1				
2016	11	3	2						1						
2016	12	1	1												
2016	13	1	1												
2016	14	1	1												
2016	15	2					2								

		230											
		70	39	7	3	4	32	26	17	19	2	7	4
		Bond formation											
**Year**	**Entry**	C(sp2)-C(sp2)	C(sp2)-C(sp3)	C(sp3)-C(sp3)	C(sp2)-C(alkyne)	C(sp2)-C(nitrile)	Csp2-N	Csp3-N	amide	Csp2-O	Csp3-O	C-Hal	sulfonamide
2016	3	1						1	1				
2016	4	1											1
2016	5	1											
2016	6		1										
2016	7	1											
2016	8	1	1										
2016	9	1	1										
2016	10								1				
2016	11	1	1					1					
2016	12		1										
2016	13		1										
2016	14	1											
2016	15						1	1					

Table S1 An assessment of 131 Fragment-to-Lead campaigns detailing i) polar fragment functionality interacting with proteins, ii) the nature of the atom growth originated from during fragment-to- lead elaboration and iii) the observed bonds formed during this process.

Total entries	131								191														230													230											
									8	0	43	25	16	7	18	41	14	9	2	1	7		149	13	0	10	18	10	4	7	4	1	0	11	3	70	39	7	3	4	32	26	17	19	2	7	4
									Fragment functionalities interacting with proteins													Nominal growing vectors														Bond formation											
Year	Entry	Fragment Hit + PDB Code (where available)	Lead + PDB Code (where available)	Institution	Target	Binding pose changed?	Scaffold Hop?	# fragment / protein interactions	Arom CH	Aliph CH	Arom N	Arom NH	Aniline NH	Aliph NH	Amide NH	CO	Acid COOH	Arom OH	Aliph OH	Arom Hal	Other polar funct.	# nominal growing vectors	Arom CH	Aliph CH	Arom N	Arom NH	Aniline NH	Aliph NH	Amide NH	CO	Acid COOH	Arom OH	Aliph OH	Arom Hal	Other polar funct.	C(sp2)-C(sp2)	C(sp2)-C(sp3)	C(sp3)-C(sp3)	C(sp2)-C(alkyne)	C(sp2)-C(nitrile)	Csp2-N	Csp3-N	amide	Csp2-O	Csp3-O	C-Hal	sulfon amide
2016	16	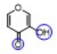	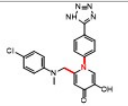	Univ Calornia, San Diego	Endonuclease	N	N	2								1		1				2	1			1											1				1						
2016	17	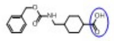	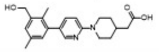	AZ	FabH	N	N	1									1																														
2016	18	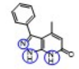	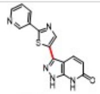 5CTY	Cubist, Evotec	GyrB	N	N	3			1	2										1	1													1											
2016	19	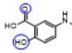	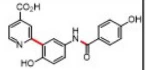 5A7W	Univ Oxford, Univ California (San Fran)	KDM4C	N	N	2									1	1				2	1						1							1							1				
2016	20	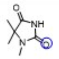 5JAH	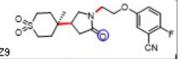 5LZ9	Astex, GSK	Lp-PLA2	N	N	1								1						2		1					1									1				1					
2016	21	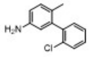 5JAO	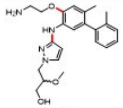 5JAU	Astex, GSK	Lp-PLA2	N	N															2	1				1														1			1			
2016	22	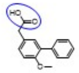 5F3T	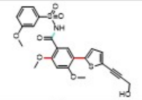 5HMZ*	Novartis, Univ Texas	RdRp	N	N	1									1					3	2								1					1							1	1			
2016	23	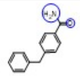 5G3M	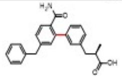 5G3N*	AZ	sPLA2	N	N	2							1	1						1	1													1											
2016	24	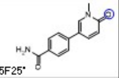 5F25*	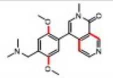 5F1H	Boehringer Ingelheim	BRD9	N	N	1								1						4	4													2								2			
2016	25	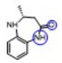 4YK0	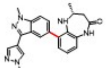 5I8G	Constellation, Genentech	CBP/EP300	N	N	2							1	1						1	1													1											
2016	26	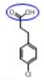 5FNQ	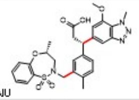 5FNU	Astex, GSK	KEAP1	N	N	1									1					2	1	1													2										
2016	27	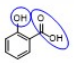 4Y03	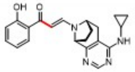 5DKH	Pfizer	PB1(5)	N	N	2									1	1				1									1					1											
2016	28	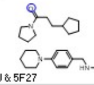 5F1J & 5F27	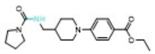 5EYR	Univ Cambridge, Institut Pasteur de Lille	EthR	N	N	1								1						1								1													1				

Table S1 An assessment of 131 Fragment-to-Lead campaigns detailing i) polar fragment functionality interacting with proteins, ii) the nature of the atom growth originated from during fragment-to- lead elaboration and iii) the observed bonds formed during this process.

Total entries		131							191														230													230											
									8	0	43	25	16	7	18	41	14	9	2	1	7		149	13	0	10	18	10	4	7	4	1	0	11	3	70	39	7	3	4	32	26	17	19	2	7	4
								Fragment functionalities interacting with proteins														Nominal growing vectors														Bond formation											
Year	Entry	Fragment Hit + PDB Code (where available)	Lead + PDB Code (where available)	Institution	Target	Binding pose changed?	Scaffold Hop?	# fragment / protein interactions	Arom CH	Aliph CH	Arom N	Arom NH	Aniline NH	Aliph NH	Amide NH	CO	Acid COOH	Arom OH	Aliph OH	Arom Hal	Other polar funct.	# nominal growing vectors	Arom CH	Aliph CH	Arom N	Arom NH	Aniline NH	Aliph NH	Amide NH	CO	Acid COOH	Arom OH	Aliph OH	Arom Hal	Other polar funct.	C(sp2)-C(sp2)	C(sp2)-C(sp3)	C(sp3)-C(sp3)	C(sp2)-C(alkyne)	C(sp2)-C(nitrile)	Csp2-N	Csp3-N	amide	Csp2-O	Csp3-O	C-Hal	sulfonamide
2017	1	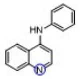	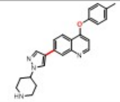	KAIST, Inha Univ, Univ Ulsan	ALK (L1196M)	N	N	1			1											1	1													1											
2017	2	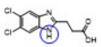	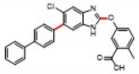 5ISO*	Merck, Metabasis	AMPK		N	1				1										2	1											1		1								1			
2017	3	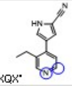 5XQX*	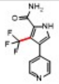 5XS2*	Roche Innovation Centre Shanghai	CDK8	Y	N	2	1							1						1	1														1										
2017	4	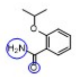 5UIQ	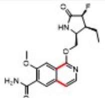 5UIU	Pfizer	IRAK4	N	N	2							1	1						2	2													2											
2017	5	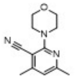 5VBO	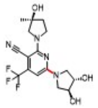 5VBZ	Pfizer	Ketohexokinase	Y	N															1	1																		1						
2017	6	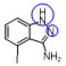	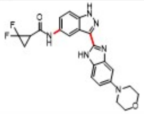	Brigham Young Univ, Tolero Pharmaceuticals, Univ Utah	PDK1	N	N	2			1	1										2	2													1					1						
2017	7	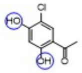	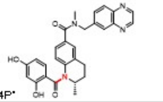 5M4P*	Vernalis	Pyruvate Dehydrogenase Kinase	N	N	2										2				1								1													1				
2017	8	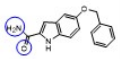 5FBI*	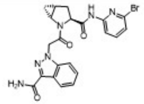 5NB7	Novartis	Complement factor D	N	Y	2							1	1																															
2017	9	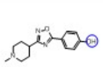 5VHC	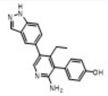	Genentech	USP7		Y	1										1																													
2017	10	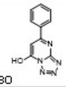 5VBO	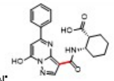 5V8N*	Pfizer, Univ Mass, Univ Hospitals Bonn	cGAS	N	N															1	1													1											

Table S1 An assessment of 131 Fragment-to-Lead campaigns detailing i) polar fragment functionality interacting with proteins, ii) the nature of the atom growth originated from during fragment-to- lead elaboration and iii) the observed bonds formed during this process.

Total entries			131						191														230													230											
									8	0	43	25	16	7	18	41	14	9	2	1	7		149	13	0	10	18	10	4	7	4	1	0	11	3	70	39	7	3	4	32	26	17	19	2	7	4
								Fragment functionalities interacting with proteins														Nominal growing vectors														Bond formation											
Year	Entry	Fragment Hit + PDB Code (where available)	Lead + PDB Code (where available)	Institution	Target	Binding pose changed?	Scaffold Hop?	# fragment / protein interactions	Arom CH	Aliph CH	Arom N	Arom NH	Aniline NH	Aliph NH	Amide NH	CO	Acid COOH	Arom OH	Aliph OH	Arom Hal	Other polar funct.	# nominal growing vectors	Arom CH	Aliph CH	Arom N	Arom NH	Aniline NH	Aliph NH	Amide NH	CO	Acid COOH	Arom OH	Aliph OH	Arom Hal	Other polar funct.	C(sp2)-C(sp2)	C(sp2)-C(sp3)	C(sp3)-C(sp3)	C(sp2)-C(alkyne)	C(sp2)-C(nitrile)	Csp2-N	Csp3-N	amide	Csp2-O	Csp3-O	C-Hal	sulfonamide
2017	11	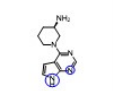 5MW3	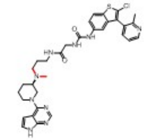 5MW4	Novartis	DOT1L	N	N	2			1	1										2						2														2					
2017	12	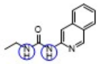	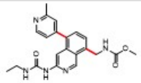 5MMO*	Actelion Pharmaceuticals	GyrB/ParE	N	N	2							2							2	2													1	1										
2017	13	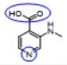	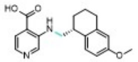 5VMP	Celgene, European Institute of Oncology, Chicago Univ	KDM4		N	2			1						1					1					1															1					
2017	14	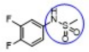 5YE8	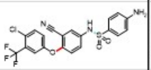 5YEA*	Chinese Academy of Science, ShanghaiTech	Lp-PLA2	N	N	1													1	2					1							1										1			1
2017	15	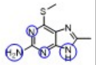	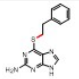 5NGS	Stockholm Univ, Karolinska Instituet, Uppsala Univ	MTH1		Y	4			2	1	1									1													1										1		
2017	16	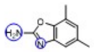	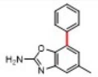 5NGT*	Stockholm Univ, Karolinska Instituet, Uppsala Univ	MTH1	N	N	2			1		1									1	1													1											
2017	17	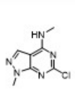 6B98	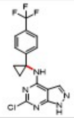 6B96	Merck	PDE2	Y	N															1					1															1					
2017	18	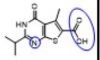	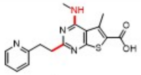 5T2Y	MIT, Univ Dundee	PglD acetyltransferase	N	N	2			1						1					2	2														1				1						
2017	19	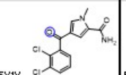 5X1V	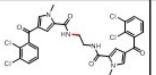 5X1W	Daiichi Sankyo	PKM2	N	N	1								1						1						1														1					
2017	20	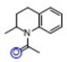 4A9H	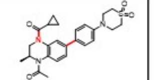 5VOM*	Forma	BRD4	N	N	1								1						2	1				1									1							1				
2017	21	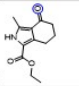 5UEP	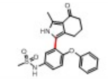 5UER	Abbvie	BRD4	Y	N	1								1						1	1													1											
2017	22	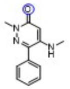 5UF0	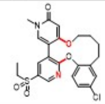 5UEX	Abbvie	BRD4	N	N	1								1						2	2																					2			

Table S1 An assessment of 131 Fragment-to-Lead campaigns detailing i) polar fragment functionality interacting with proteins, ii) the nature of the atom growth originated from during fragment-to- lead elaboration and iii) the observed bonds formed during this process.

Total entries		131							191														230													230											
									8	0	43	25	16	7	18	41	14	9	2	1	7		149	13	0	10	18	10	4	7	4	1	0	11	3	70	39	7	3	4	32	26	17	19	2	7	4
								Fragment functionalities interacting with proteins														Nominal growing vectors														Bond formation											
Year	Entry	Fragment Hit + PDB Code (where available)	Lead + PDB Code (where available)	Institution	Target	Binding pose changed?	Scaffold Hop?	# fragment / protein interactions	Arom CH	Aliph CH	Arom N	Arom NH	Aniline NH	Aliph NH	Amide NH	CO	Acid COOH	Arom OH	Aliph OH	Arom Hal	Other polar funct.	# nominal growing vectors	Arom CH	Aliph CH	Arom N	Arom NH	Aniline NH	Aliph NH	Amide NH	CO	Acid COOH	Arom OH	Aliph OH	Arom Hal	Other polar funct.	C(sp2)-C(sp2)	C(sp2)-C(sp3)	C(sp3)-C(sp3)	C(sp2)-C(alkyne)	C(sp2)-C(nitrile)	Csp2-N	Csp3-N	amide	Csp2-O	Csp3-O	C-Hal	sulfonamide
2017	23	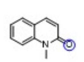 5T4U	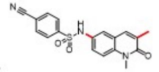 5T4V*	UCL, SGC, Pfizer, CRUK, AZ etc	BRPF	N	N	1								1						2	2														1				1						
2017	24	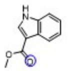 6AXQ	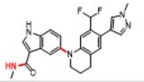 6AY3	Genentech, Wuxi, Editas Medicine	CBP/P300	N	N	1								1						2	1							1											1		1				
2017	25	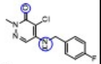 5MKX*	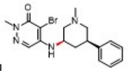 5MLJ	GSK, Cellzome, Univ Strathclyde	PCAF/GCN5	N	N	2					1			1						1					1															1					
2017	26	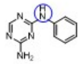 5X4M	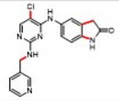 5X4Q	Takeda	BCL6	N	N	1					1									4	3				1										1				1	1				1	
2017	27	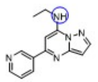 5N1X	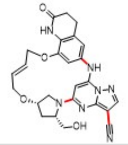 5N1Z	AZ, Pharmaron	BCL6	Y	N	1					1									3	2				1													1	2						
2017	28	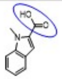	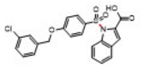	Dalian University of technology	Mcl-1	N	N	1									1					1				1																					1
2017	29	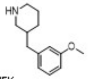 5U5K	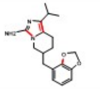 5U62*	Novartis	PRC2/EED	N	N															3	1	1				1									1				1			1			
2017	30	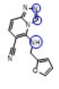 5H24	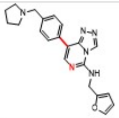	Novartis	PRC2/EED	N	N	3	1		1		1									1	1													1											
2018	1	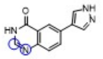	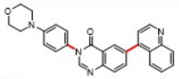 6GIN	ICR, Univ Barcelona	ALK2	N	N	2	1		1											2	1			1										1					1						
2018	2	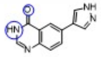	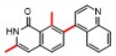 6GIP	ICR, Univ Barcelona	ALK2	Y	N	2				1				1						3	3													1	2										
2018	3	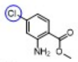 3MS9	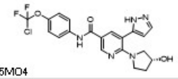 5MO4	Novartis	BCR-ABL1	N	Y	1												1																											
2018	4	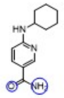 6DI0	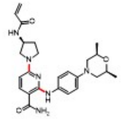 6DI1*	EMD Serono	BTK	N	N	2							1	1						2	2																		2						

Table S1 An assessment of 131 Fragment-to-Lead campaigns detailing i) polar fragment functionality interacting with proteins, ii) the nature of the atom growth originated from during fragment-to- lead elaboration and iii) the observed bonds formed during this process.

Total entries			131						191														230													230											
									8	0	43	25	16	7	18	41	14	9	2	1	7		149	13	0	10	18	10	4	7	4	1	0	11	3	70	39	7	3	4	32	26	17	19	2	7	4
								Fragment functionalities interacting with proteins														Nominal growing vectors														Bond formation											
Year	Entry	Fragment Hit + PDB Code (where available)	Lead + PDB Code (where available)	Institution	Target	Binding pose changed?	Scaffold Hop?	# fragment / protein interactions	Arom CH	Aliph CH	Arom N	Arom NH	Aniline NH	Aliph NH	Amide NH	CO	Acid COOH	Arom OH	Aliph OH	Arom Hal	Other polar funct.	# nominal growing vectors	Arom CH	Aliph CH	Arom N	Arom NH	Aniline NH	Aliph NH	Amide NH	CO	Acid COOH	Arom OH	Aliph OH	Arom Hal	Other polar funct.	C(sp2)-C(sp2)	C(sp2)-C(sp3)	C(sp3)-C(sp3)	C(sp2)-C(alkyne)	C(sp2)-C(nitrile)	Csp2-N	Csp3-N	amide	Csp2-O	Csp3-O	C-Hal	sulfonamide
2018	5	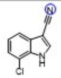	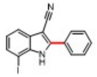	Technische Univ Braunschweig, RWTH Aachen, ManRos	DYRK1A	N	N	1													1	1	1													1											
2018	6	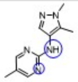 6G92	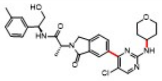 6G9N	Astex	ERK1/2	N	N	2			1		1									2	1				1									1						1					
2018	7	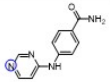 6CJE*	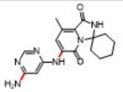 6CK6	eFFector Therapeutics et al	MNK1/2	N	N	1			1											2	1				1														2						
2018	8	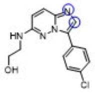	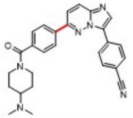	A*Star et al	MNK1/2	N	N	2	1		1											1	1													1											
2018	9	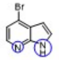	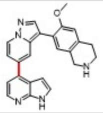	A*STAR	PKC iota	N	N	2			1	1										1												1		1											
2018	10	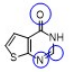	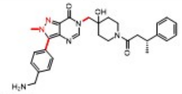 5N9R*	Almac, Queen's Univ Belfast	USP7		N	3	1		1					1						3	1			2										1						2					
2018	11	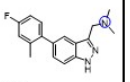 5O4V	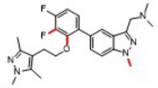 5MU6	Imperial	Human N-myristoyltransferase	N	N	1						1								3	2			1																1		1		1	
2018	12	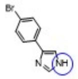 5OU2	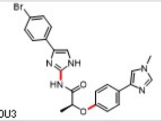 5OU3	Univ Cambridge, Univ Cape Town, Univ Melbourne	IMPDH	N	N	1				1										2	1											1							1			1			
2018	13	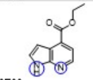 6F20	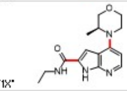 6F1X*	Sprint Bioscience et al	MTH1	N	N	2			1	1										2	2													1					1						
2018	14	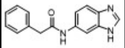	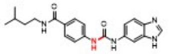	Abbvie	NAMPT	N	N															1								1													1				
2018	15	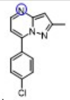 5XUJ	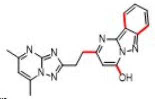 5XUI*	Astellas	PDE10A	N	N	1			1											4	4													2	1							1			

Table S1 An assessment of 131 Fragment-to-Lead campaigns detailing i) polar fragment functionality interacting with proteins, ii) the nature of the atom growth originated from during fragment-to- lead elaboration and iii) the observed bonds formed during this process.

Total entries			131						191														230													230											
									8	0	43	25	16	7	18	41	14	9	2	1	7		149	13	0	10	18	10	4	7	4	1	0	11	3	70	39	7	3	4	32	26	17	19	2	7	4
								Fragment functionalities interacting with proteins														Nominal growing vectors														Bond formation											
Year	Entry	Fragment Hit + PDB Code (where available)	Lead + PDB Code (where available)	Institution	Target	Binding pose changed?	Scaffold Hop?	# fragment / protein interactions	Arom CH	Aliph CH	Arom N	Arom NH	Aniline NH	Aliph NH	Amide NH	CO	Acid COOH	Arom OH	Aliph OH	Arom Hal	Other polar funct.	# nominal growing vectors	Arom CH	Aliph CH	Arom N	Arom NH	Aniline NH	Aliph NH	Amide NH	CO	Acid COOH	Arom OH	Aliph OH	Arom Hal	Other polar funct.	C(sp2)-C(sp2)	C(sp2)-C(sp3)	C(sp3)-C(sp3)	C(sp2)-C(alkyne)	C(sp2)-C(nitrile)	Csp2-N	Csp3-N	amide	Csp2-O	Csp3-O	C-Hal	sulfonamide
2018	16	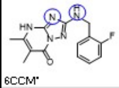 6CCM*	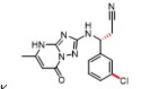 6CCK	Novartis	PPAT	N	N	2			1		1									2	1	1														1								1	
2018	17	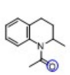 4A9H	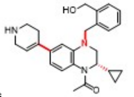 6FFG	GSK, Strathclyde	BET family BD2	N	N	1								1						3	1	1			1									1		1				1					
2018	18	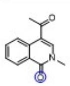	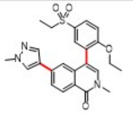 6CKS	Celgene Quanticel Research, Univ Chicago	BRD4-BD1	N	N	1								1						2	2													2											
2018	19	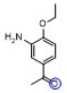 4TQN*	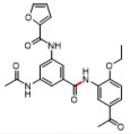 5NLK	Univ Zurich	CBP	Y	N	1								1						1					1																1				
2018	20	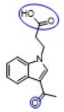 5XXH*	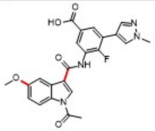	Chinese Academy of Sciences	CBP/EP300	N	N	2								1	1					2	2													1								1			
2018	21	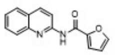 5OCO*	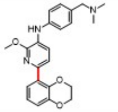 6FA4	Univ Oxford, Univ Leeds et al	HRAS	N	Y															1	1													1											

Table S1 An assessment of 131 Fragment-to-Lead campaigns detailing i) polar fragment functionality interacting with proteins, ii) the nature of the atom growth originated from during fragment-to- lead elaboration and iii) the observed bonds formed during this process.

2018	22	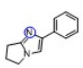 6D9X	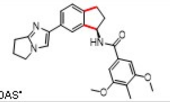 6DAS*	Vanderbilt	WDR5	N	N	1			1											2	2														2										
2018	23	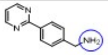	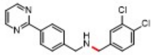	Vrije Univ et al	β2AR		N	1						1								1						1														1					
2018	24	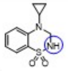 4N07	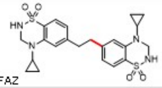 6FAZ	Univ Copenhagen et al	AMPA receptor	N	N	1													1	1	1														1										
2018	25	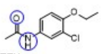 6FZU	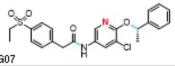 6G07	Novartis	RORγT	N	N	2							1	1						2					1					1											1		1		
2018	26	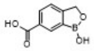	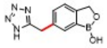	GSK, Cellzome	Unknown		N															1	1														1										

Total entries	131

Year	Entry	Fragment Hit + PDB Code (where available)	Lead + PDB Code (where available)	Institution	Target	Binding pose changed?	Scaffold Hop?
2019	1	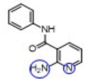	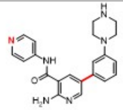 6ILZ	A*STAR	PKC-ι	N	N
2019	2	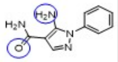 6SZE	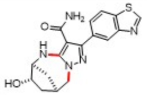 6UL8	GSK	RIP2	Y	N
2019	3	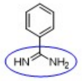	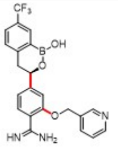 6SKB*	GSK, INSERM	Kallikrein 5 (KLK5)	N	N
2019	4	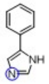 2D0T	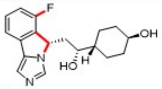 6O3I	NewLink Genetics, Genentech	IDO1	N	N
2019	5	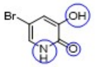 4MK1	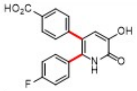 6NEL	State Univ New Jersey, Univ Rochester Medical Center	Influenza A endonuclease	N	N
2019	6	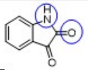 2XFP	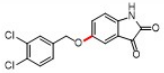	Hefei Univ Technology	hMAO-B		N
2019	7	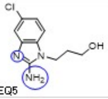 6EQ5	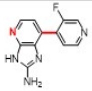 6EQ7	Univ Zurich, Univ Applied Sciences and Arts Northwestern Switzerland	MTH1	Y	N
2019	8	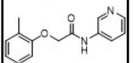 6R8P	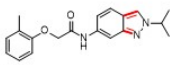 6R8Q*	Univ College, London; Univ Oxford; The Francis Crick Institute	Notum	N	N
2019	9	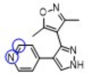 6G25	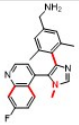 6G2O	Boehringer Ingelheim, Univ Toronto, Univ Oxford, Univ North Carolina, Cold Spring Harbor	NSD3-PWWP1	N	N
2019	10	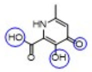 6E6V	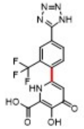 6E6W*	UCSD, Nankai Univ	PA_N Endonuclease (Influenza virus)	N	N

Fragment functionalities interacting with proteins (191)

Entry	# fragment / protein interactions	Arom CH	Aliph CH	Arom N	Arom NH	Aniline NH	Aliph NH	Amide NH	CO	Acid COOH	Arom OH	Aliph OH	Arom Hal	Other polar funct.
Totals		8	0	43	25	16	7	18	41	14	9	2	1	7
1	2			1		1								
2	2					1			1					
3	1													1
4	1			1										
5	3				1				1		1			
6	2							1	1					
7	2			1		1								
8														
9	2	1		1										
10	3								1	1	1			

Nominal growing vectors (230)

Entry	# nominal growing vectors	Arom CH	Aliph CH	Arom N	Arom NH	Aniline NH	Aliph NH	Amide NH	CO	Acid COOH	Arom OH	Aliph OH	Arom Hal	Other polar funct.
Totals		149	13	0	10	18	10	4	7	4	1	0	11	3
1	1	1												
2	2	1			1									
3	2	2												
4	3	2			1									
5	2	1											1	
6	1	1												
7	1	1												
8	2	2												
9	4	3			1									
10	1	1												

Bond formation (230)

Entry	C(sp2)-C(sp2)	C(sp2)-C(sp3)	C(sp3)-C(sp3)	C(sp2)-C(alkyne)	C(sp2)-C(nitrile)	Csp2-N	Csp3-N	amide	Csp2-O	Csp3-O	C-Hal	sulfon amide
Totals	70	39	7	3	4	32	26	17	19	2	7	4
1	1											
2						1	1					
3		1							1			
4		1					1				1	
5	2											
6									1			
7	1											
8	1					1						
9	3					1						
10	1											

Table S1 An assessment of 131 Fragment-to-Lead campaigns detailing i) polar fragment functionality interacting with proteins, ii) the nature of the atom growth originated from during fragment-to- lead elaboration and iii) the observed bonds formed during this process.

Table S1 An assessment of 131 Fragment-to-Lead campaigns detailing i) polar fragment functionality interacting with proteins, ii) the nature of the atom growth originated from during fragment-to- lead elaboration and iii) the observed bonds formed during this process.

Total entries			131						191														230													230											
									8	0	43	25	16	7	18	41	14	9	2	1	7		149	13	0	10	18	10	4	7	4	1	0	11	3	70	39	7	3	4	32	26	17	19	2	7	4
								Fragment functionalities interacting with proteins														Nominal growing vectors														Bond formation											
Year	Entry	Fragment Hit + PDB Code (where available)	Lead + PDB Code (where available)	Institution	Target	Binding pose changed?	Scaffold Hop?	# fragment / protein interactions	Arom CH	Aliph CH	Arom N	Arom NH	Aniline NH	Aliph NH	Amide NH	CO	Acid COOH	Arom OH	Aliph OH	Arom Hal	Other polar funct.	# nominal growing vectors	Arom CH	Aliph CH	Arom N	Arom NH	Aniline NH	Aliph NH	Amide NH	CO	Acid COOH	Arom OH	Aliph OH	Arom Hal	Other polar funct.	C(sp2)-C(sp2)	C(sp2)-C(sp3)	C(sp3)-C(sp3)	C(sp2)-C(alkyne)	C(sp2)-C(nitrile)	Csp2-N	Csp3-N	amide	Csp2-O	Csp3-O	C-Hal	sulfonamide
2019	11	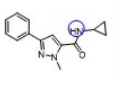 6RIH	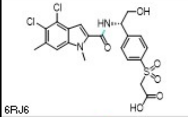 6RJ6	Boehringer Ingelheim, Shanghai ChemPartner	PHGDH	N	N	1							1							1								1													1				
2019	12	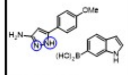 6QOT & 6QOU	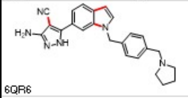 6QR6	Univ Cambridge, Royal Papworth Hospital, National Institutes of [illegible]	*Mab* TrmD	N	N	2			1	1										3	3													1				1	1						
2019	13	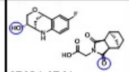 6R9S & 6RA1	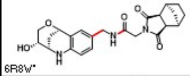 6R8W*	KGaA, EMD Serono, Edelris, Proteros Biostructures	Cyclophilin D	N	N	2								1			1			2									1			1			1						1				
2019	14	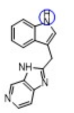 4EPV	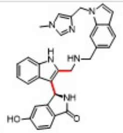 6GJ7*	Boehringer Ingelheim, Vanderbilt Univ	K-Ras^{G12D}	N	N	1				1										2	2														2										
2019	15	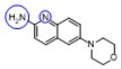	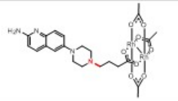	Rice Univ	Lyn SH3	N	N	2			1		1									1						1														1					
2019	16	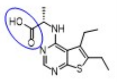	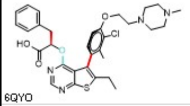 6QYO	Servier, Vernalis	Mcl-1	N	N	1									1					2	1	1												1	1										
2019	17	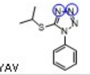 5YAV	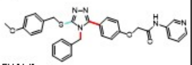 5YAW*	Shanghai Institute of Materia Medica	PDE5	N	N	2			2											3	2												1	1						1		1			
2019	18	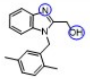 6OOY	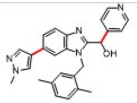 6OP0	UCB, Covance, Broad Institute	TNF	N	N	2			1								1			2	1	1												1	1										
2019	19	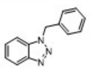	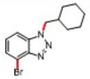	Richter Plc., Hungarian Academy of Sciences, Mitsubishi Tanabe	mGluR2		N															2	1			1																1				1	
2019	20	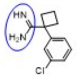 6NCN	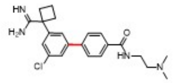 6NCO*	AbbVie	Apolipoprotein E4	N	N	1													1	1	1													1											

*** PDB code is for a fragment or hit related structure**

Footnote 1: Dockings were used in place of structures for: A) Hit: 2015-27, 2016-7, 2016-8, 2016-19, 2017-15, 2017-16, 2017-18, 2018-1, 2018-2, 2018-14, 2018-18, 2019-1; B) Lead: 2015-21; C) 2015-9, 2015-13, 2015-17, 2016-1, 2016-5, 2016-16, 2017-1, 2017-6, 2018-5, 2018-9, 2018-20

Footnote 2: No structural information available for: A) Hit: 2017-2, 2017-13,2019-15; B) Lead: 2015-24, 2016-4, 2019-6; C) Both: 2018-10, 2018-23, 2018-26, 2019-19
